# The Role of DNA Methylation in Genome Defense in Cnidaria and Other Invertebrates

**DOI:** 10.1093/molbev/msac018

**Published:** 2022-01-27

**Authors:** Hua Ying, David C Hayward, Alexander Klimovich, Thomas C G Bosch, Laura Baldassarre, Teresa Neeman, Sylvain Forêt, Gavin Huttley, Adam M Reitzel, Sebastian Fraune, Eldon E Ball, David J Miller

**Affiliations:** 1 Research School of Biology, Australian National University, Canberra, ACT, Australia; 2 Zoological Institute, Christian Albrechts University, Kiel, Germany; 3 Collaborative Research Center for the Origin and Function of Metaorganisms, Christian Albrechts University, Kiel, Germany; 4 Department of Zoology and Organismal Interactions, Heinrich-Heine-University, Düsseldorf, Germany; 5 Biological Data Institute, Australian National University, Canberra, ACT, Australia; 6 ARC Centre of Excellence for Coral Reef Studies, Australian National University, Canberra, ACT, Australia; 7 Department of Biological Sciences, University of North Carolina, Charlotte, NC, USA; 8 ARC Centre of Excellence for Coral Reef Studies, James Cook University, Townsville, QLD, Australia; 9 College of Public Health, Medical and Veterinary Sciences, James Cook University, Townsville, QLD, Australia; 10 Centre for Tropical Bioinformatics and Molecular Biology, James Cook University, Townsville, QLD, Australia; 11 Marine Climate Change Unit, Okinawa Institute of Science and Technology, Onna, Japan

**Keywords:** DNA methylation, epigenetics, transposons, Cnidaria, coral, genome defense

## Abstract

Considerable attention has recently been focused on the potential involvement of DNA methylation in regulating gene expression in cnidarians. Much of this work has been centered on corals, in the context of changes in methylation perhaps facilitating adaptation to higher seawater temperatures and other stressful conditions. Although first proposed more than 30 years ago, the possibility that DNA methylation systems function in protecting animal genomes against the harmful effects of transposon activity has largely been ignored since that time. Here, we show that transposons are specifically targeted by the DNA methylation system in cnidarians, and that the youngest transposons (i.e., those most likely to be active) are most highly methylated. Transposons in longer and highly active genes were preferentially methylated and, as transposons aged, methylation levels declined, reducing the potentially harmful side effects of CpG methylation. In Cnidaria and a range of other invertebrates, correlation between the overall extent of methylation and transposon content was strongly supported. Present transposon burden is the dominant factor in determining overall level of genomic methylation in a range of animals that diverged in or before the early Cambrian, suggesting that genome defense represents the ancestral role of CpG methylation.

## Introduction

In metazoans, DNA methylation occurs predominantly as methylated cytosine residues in CpG (mCpG) motifs, and was first characterized four decades ago as a repressive epigenetic marker that regulates gene expression and cell differentiation (Holliday and Pugh 1975; Compere and Palmiter 1981; Lieberman et al. 1983) in vertebrates. Methylation at CpG motifs is one characteristic of transcriptionally silent chromatin, including heterochromatin and, for promoters of many vertebrate genes that are expressed in a tissue-specific manner, transcriptional activity is inversely related to promoter methylation. Removal of methylation marks, executed by enzymes known as ten-eleven translocation methylcytosine dioxygenases (TET enzymes), is necessary but not sufficient to permit transcription (Mariani et al. 2014).

Because the CpG methylation system has been lost from *Caenorhabditis*, and its highly diverged equivalent in *Drosophila* went unrecognized until 1999 (Tweedie et al. 1999), it was initially assumed that CpG methylation was significant only in vertebrates (Bird et al. 1995). However, this assumption proved to be incorrect, as the genomes of a phylogenetically diverse range of invertebrates, including representatives of early diverging arthropod lineages, are now known to be substantially methylated (Wang et al. 2006; Lewis et al. 2020).

Data from sponges (de Mendoza et al. 2019) indicate that the DNA methylation machinery was present in the metazoan common ancestor, and the system appears to have been largely conserved between animal phyla. However, in general, vertebrates and invertebrates differ with respect to the extent and nature of CpG modification. In mammals, the majority (>70%) of cytosines in CpG motifs are typically methylated, and genome methylation levels (GMLs) are high. Invertebrate genomes are generally much more sparsely methylated, the methylation landscape often being described as “mosaic-like” (Feng et al. 2010; Zemach et al. 2010). Early studies of 29 animals (Grunau et al. 2001; Gregory 2010; Lechner et al. 2013) suggested that GML of 40% is the boundary between invertebrate (<40%) and vertebrate (>40%) genomes (Lechner et al. 2013) and this generalization still largely holds (Lewis et al. 2020). In invertebrates, promoters are usually not methylated and most of the mCpGs are concentrated on gene bodies, especially at actively transcribed regions, a phenomenon frequently referred to as gene body methylation (gbM) (Sarda et al. 2012; Keller et al. 2016). The consensus is therefore that, in invertebrates, DNA methylation is not directly associated with repressing transcription, but primarily has other regulatory roles.

In addition to the well-established role of CpG methylation in regulating gene expression, transposons are extensively methylated in mammalian genomes (Bestor and Tycko 1996; Bestor et al. 2015). The observation that transposons are targets of DNA methylation (Matzke et al. 1989), along with the common features of bacterial and eukaryotic DNA methyltransferases (DNMTs), suggesting a common origin (Bestor et al. 1988), led Bestor (1990) to propose the genome defense hypothesis. According to this hypothesis, eukaryotic DNA methylation systems evolved from bacterial “immune” (restriction–modification) systems, acquiring an analogous role in silencing parasitic sequences (e.g., transposons) via methylation (Yoder et al. 1997). This function allowed the genome to manage transposable element (TE) activity and become tolerant to TE invasions, which led to genome expansion. Hence, DNA methylation was predicted to be correlated with genomic parasite (TE) load and genome size (Regev et al. 1998). Although recent research supports this relationship in vertebrates (Zhou et al. 2020), in the case of invertebrates, this hypothesis has been rejected outright (Regev et al. 1998) due to the lack of correlation between DNA methylation level (ML) and genome size. Moreover, transposons in invertebrate genomes were found to be depleted of DNA methylation, and consequently are generally considered not to be specifically targeted by DNA methylation (de Mendoza et al. 2019). Rather, Regev et al. (1998) interpreted the coexistence of DNA methylation and high cell turnover as evidence that methylation plays primarily regulatory roles in invertebrates. If this is the case, a natural question is whether changes in GML are associated with phenotypic plasticity. Bewick et al. (2017) studied a wide range of insects and found no evidence for evolutionary association between DNA methylation and sociality. Roberts and Gavery (2012) suggested that limited methylation changes in exons could facilitate differential transcript expression in highly fluctuating environments. However, based on the available evidence, it is difficult to reconcile the idea that methylation serves such regulatory roles with the observation that GML varies >10-fold across the invertebrates (Roberts and Gavery 2012).

Methylome studies of nonbilaterian metazoans can provide insights to understand the evolutionary origin and associated functions of DNA methylation in animals. Previous studies in this area have been predominantly on corals, which have received a disproportionate amount of attention in the past few years due to interest in the potential role of methylation and other epigenetic mechanisms in adapting to a rapidly changing climate. Analyses of CpG depletion (Dixon et al. 2014; Dimond and Roberts 2016) or bisulfite sequencing (BS-Seq) (Zemach et al. 2010) data implied that as in other invertebrates, housekeeping genes were highly methylated relative to differentially expressed genes in cnidarian genomes. These genes have low rates of evolution and show greater codon bias (Dixon et al. 2016) and less transcriptional noise (variation in expression levels) (Liew et al. 2018).

Both genetic (e.g., developmental differences) and environmental factors appear to contribute to differences in methylation patterns in cnidarians. Many stimuli have been analyzed for their effect on patterns of methylation including thermal stress (Dimond and Roberts 2016; Durante et al. 2019), acidification (Dimond and Roberts 2016; Putnam et al. 2016; Liew et al. 2018), transplantation to a common garden (Dimond and Roberts 2020), reciprocal transplantation (Dixon et al. 2014, 2018), and seasonal changes (Rodríguez-Casariego et al. 2020). Although these treatments often resulted in global changes in both gbM and gene expression patterns, correlation between these was either very limited or absent, suggesting that CpG methylation may have other roles in cnidarians.

To better understand the extent and significance of DNA methylation in early diverging eumetazoans, we investigated methylation patterns in the genomes of three members of the phylum Cnidaria, including two representatives of the earliest diverging class Anthozoa—the sea-anemone *Nematostella vectensis* (hereafter *Nematostella*) and the coral *Acropora millepora* (hereafter *Acropora*) and *Hydra vulgaris* (originally published as *H. magnipapillata* and referred to hereafter as *Hydra*), a member of the later diverging class Hydrozoa. These species are among the most studied members of the phylum and provide high-quality data sets for studying patterns of methylation. *Acropora* (*millepora*) has been among the most-studied species of corals (Dixon et al. 2014, 2016, 2018; Dimond and Roberts 2016; Ball et al. 2021; Dixon and Matz 2021). The results imply that, although additional regulatory roles are likely exaptations, transposons are the primary targets of DNA methylation in cnidarians. This model explains both the enormous (>10-fold) variation in GML, and the high level of conservation of the machinery responsible for transcriptional silencing observed across the invertebrates.

## Results

### The Methylation Landscapes of Representative Cnidarians

**Fig. 1.**
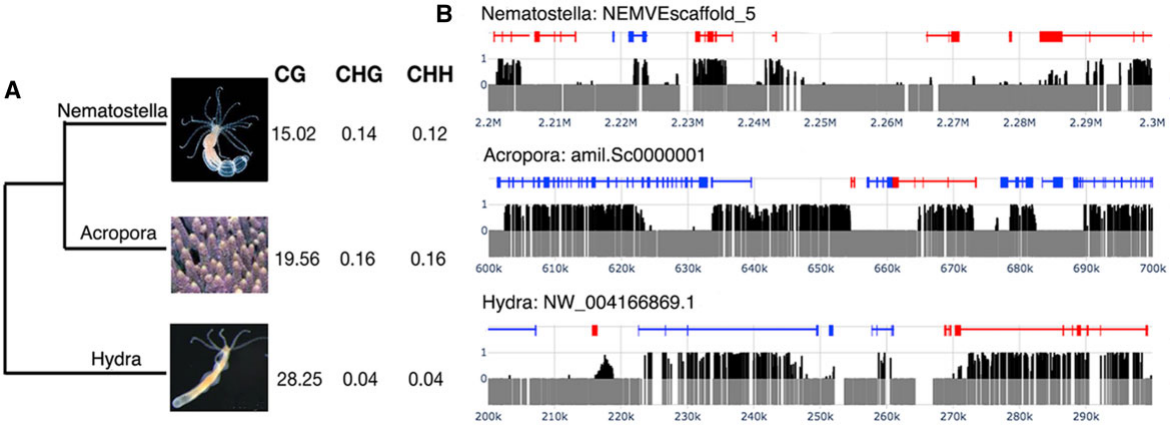
General characteristics of the methylomes of *Nematostella*, *Acropora*, and *Hydra*. (*A*) The cladogram at left summarizes relationships between the three cnidarians studied here, and the table indicates the occurrence of 5-methylcytosine in different sequence contexts in the three cnidarians. (*B*) mCpG is patchily distributed across the genomes of *Nematostella*, *Acropora*, and *Hydra*. The figure shows the distribution of CpG (gray vertical bars) and mCpG (black vertical bars) across typical regions of the genomes of *Nematostella* (upper), *Acropora* (middle), and *Hydra* (lower). The positions of genes on the + and −strands are indicated by the blue and red lines, respectively; boxes show the positions of exons.

Genome assemblies are now available for a number of cnidarians, but methylome data are available for only a small subset of these (supplementary table S1, Supplementary Material online), one consequence of which is that general patterns are unclear. To investigate cnidarian DNA methylation patterns, whole-genome BS-Seq data were generated from *Nematostella, Acropora*, and *Hydra—*three cnidarians that are well-represented in the databases (supplementary table S2, Supplementary Material online). At a genome-wide level, 15.0%, 19.6%, and 28.3% of CpG dinucleotides were methylated (denoted as mCpG) in *Nematostella*, *Acropora*, and *Hydra*, respectively (fig. 1*A* and supplementary table S3, Supplementary Material online). DNA methylation in CHG and CHH (H = A, G, or T) contexts was barely detectable, implying that these are likely to actually be methylation-free and thus supporting a very low false discovery rate at CpG motifs. Meanwhile, the DNA methylation landscapes of these cnidarians resemble those of many other invertebrates in that methylation is sparse and patchily distributed (fig. 1*B*). Genome-wide DNA MLs in cnidarians (6.37–28.3% including *Exaiptasia pallida*; Li et al. 2018 and *Stylophora pistillata*; Liew et al. 2018) are broadly in line with those observed in arthropods (3–33%) (see below), suggesting that high variation in DNA ML is a common phenomenon in different phyla.

### Transposons Are Targets of DNA Methylation in Cnidaria

**Fig. 2.**
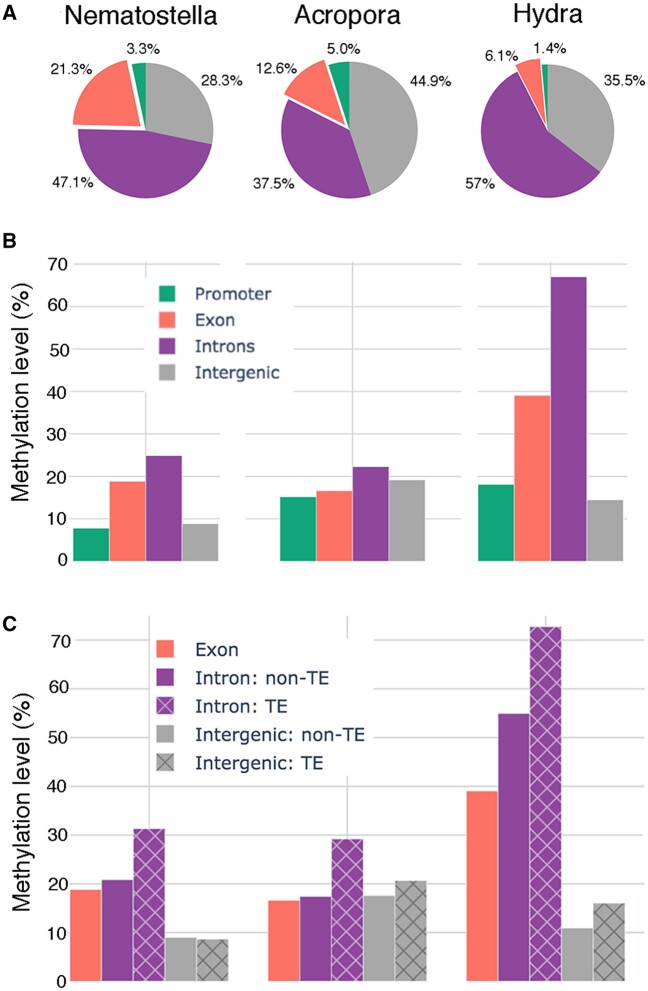
(*A*) The distribution of mCpG in introns, exons, promoters, and intergenic regions across the genomes of the three cnidarians. Percentages were calculated as 100×number of mCpG in a genomic region/total number of mCpG in the genome. (*B*) Introns are more heavily methylated than exons, promoters or intergenic regions in all three cnidarians studied here. Methylation levels (ML%) were calculated as 100×number of mCpG/total number of CpG (coverage ≥2) in each genomic region. (*C*) Intronic transposons are methylated above the (intronic) background across all three cnidarians and in both *Acropora* and *Hydra* (but not *Nematostella*) transposons in intergenic regions are methylated to a higher level than the corresponding (nonrepetitive) background.

It has been reported that DNA methylation preferentially targets intragenic regions in invertebrates (Feng et al. 2010; Zemach et al. 2010), a pattern often referred to as gene body methylation (gbM). Although in the three cnidarians examined here, 50.1–68.4% of mCpGs occurred within the gene body, these occurred predominantly in introns rather than exons. Since genomic features are rarely evenly distributed, DNA MLs (defined as the ratio of mCpGs to covered CpGs; ≥2 reads), were calculated for different genomic features. This analysis confirmed that CpGs in introns were more frequently methylated than were those in exons, whereas promoters and intergenic regions were relatively depleted of DNA methylation (fig. 2*A* and *B*). Similar findings have been reported for two other cnidarians, *Stylophora* (Liew et al. 2018) and *Exaiptasia* (Li et al. 2018). Within introns, repetitive sequences, the vast majority of which were transposons (supplementary table S4, Supplementary Material online), were methylated significantly more frequently (odds ratios [OR] ranged from 1.76 to 2.19, *P* < 10^−16^; supplementary table S5, Supplementary Material online) than were nonrepetitive sequences, which were more frequently methylated than exons (fig. 2*C*). This was observed irrespective of repeat classes. This contrast is most obvious in *Hydra*, in which intronic repetitive sequences were hypermethylated to a vertebrate-like level (72.76%), whereas only 39.05% exonic CpGs were methylated (fig. 2*C*). Additionally, within introns, repetitive sequences were enriched in both CpG dinucleotides and mCpGs over the nonrepetitive background. For example, in *Acropora*, transposons accounted for 32% of intron sequences, but comprised 42% and 54% of intronic CpG dinucleotides and mCpGs, respectively. Similar phenomena were observed in both *Nematostella* and *Hydra* (supplementary table S5, Supplementary Material online). These results indicate that intronic transposons are preferentially targeted by DNA methylation.

Despite intergenic regions harboring lower densities of mCpGs than the gene body, specific transposon classes were apparently targeted by DNA methylation in the three species. Overall, both *Acropora* (OR 1.22 ± 0.01) and *Hydra* (OR 1.56 ± 0.01) showed the same pattern of mCpG enrichment of repetitive sequences compared with the nonrepeat background in intergenic regions, whereas *Nematostella* (OR 0.91 ± 0.01) did not. In the case of *Acropora*, intergenic regions were more highly methylated (19.16%) than exons (16.61%). Retrotransposons comprised 56.78% of the known transposon complement, and those in intergenic regions had a similar ML (28.64%) to their intronic counterparts (31.44%), supporting transposons as the main targets of methylation. Intergenic (nontransposon) background DNA MLs were similar in *Hydra* and *Nematostella* (10.92% and 9.00%, respectively), whereas gene body methylation was much higher (fig. 2*C*) for *Hydra*, and such a trend was clearly observed for both retrotransposons (ML 15.68%, OR 1.52 ± 0.01) and DNA transposons (ML 17.42%, OR 1.72 ± 0.01). In the *Nematostella* genome, on the other hand, intergenic DNA transposons (which comprise over 60% of known transposons in this species) were undermethylated (ML 8.22%, OR 0.91 ± 0.01), but retrotransposons (ML 11.31%, OR 1.29 ± 0.01) were significantly more highly methylated (*P* < 10^−16^) than the genome background (supplementary table S6, Supplementary Material online).

### Young Transposons Are the Primary Targets of Methylation

**Fig. 3.**
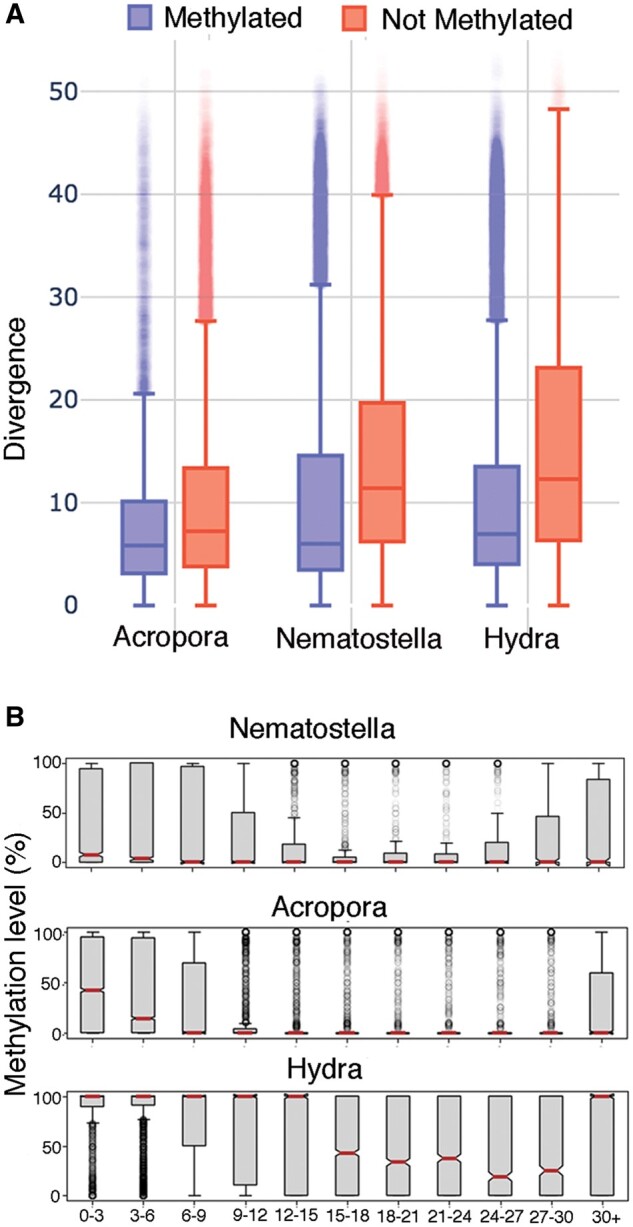
(*A*) Methylated transposons are significantly younger than unmethylated transposons in all three cnidarians. The figure shows box plots for the three cnidarian species in which the color represents methylated (blue, ML≥10%) or unmethylated (red, ML<10%) transposons. The *y* axis represents the Kimura distance (divergence) distribution. In each case, the *P* value for comparison of the two distributions was <10^−16^. (*B*) DNA ML distribution of intronic transposons across different divergence bins.

The relationship between transposon age and the extent of DNA methylation was investigated by using RepeatMasker (see Materials and Methods) to estimate the divergence (Kimura distance) of individual transposon sequences compared with the inferred consensus for each transposon family. Low levels of divergence indicate sequences that have accumulated few mutations from their common ancestor and therefore are considered to be young (Giordano et al. 2007; Quesneville 2020). Interestingly, the *Nematostella* genome hosts a much higher proportion of young transposons (63.83% with divergence <10) than do those of *Acropora* (44.39%) or *Hydra* (38.87%) (supplementary fig. S1, Supplementary Material online). Despite these differences, when transposons (covered CpG ≥ 10) were classified as either unmethylated (ML ≤ 10%) or methylated (ML > 10%), the divergence of methylated transposons was found to be significantly lower than those of unmethylated transposons (Mann–Whitney *U* test, *P* < 10^−16^, fig. 3*A*). Further analysis revealed a negative trend between levels of DNA methylation and divergence when divergence was below 30. This trend was observed for both intronic (fig. 3*B*) and intergenic (supplementary fig. S2 and table S7, Supplementary Material online) transposons in all three cnidarian genomes. Therefore, DNA methylation preferentially targets young transposons which are presumed to be the most active transposon group in the genome. However, the most diverged (oldest) transposon group (divergence 30+) showed unexpectedly high MLs in each of the three cnidarians (fig. 3*B* and supplementary fig. S2, Supplementary Material online); this group represents only a small proportion (5–12%) of transposons, but these are worth further investigation as they may have coevolved with the host genome into regulatory roles (see, for example, Cosby et al. [2019]).

### Transposon Methylation Is a Major Contributor to Increased gbM with Gene Expression Level

**Fig. 4.**
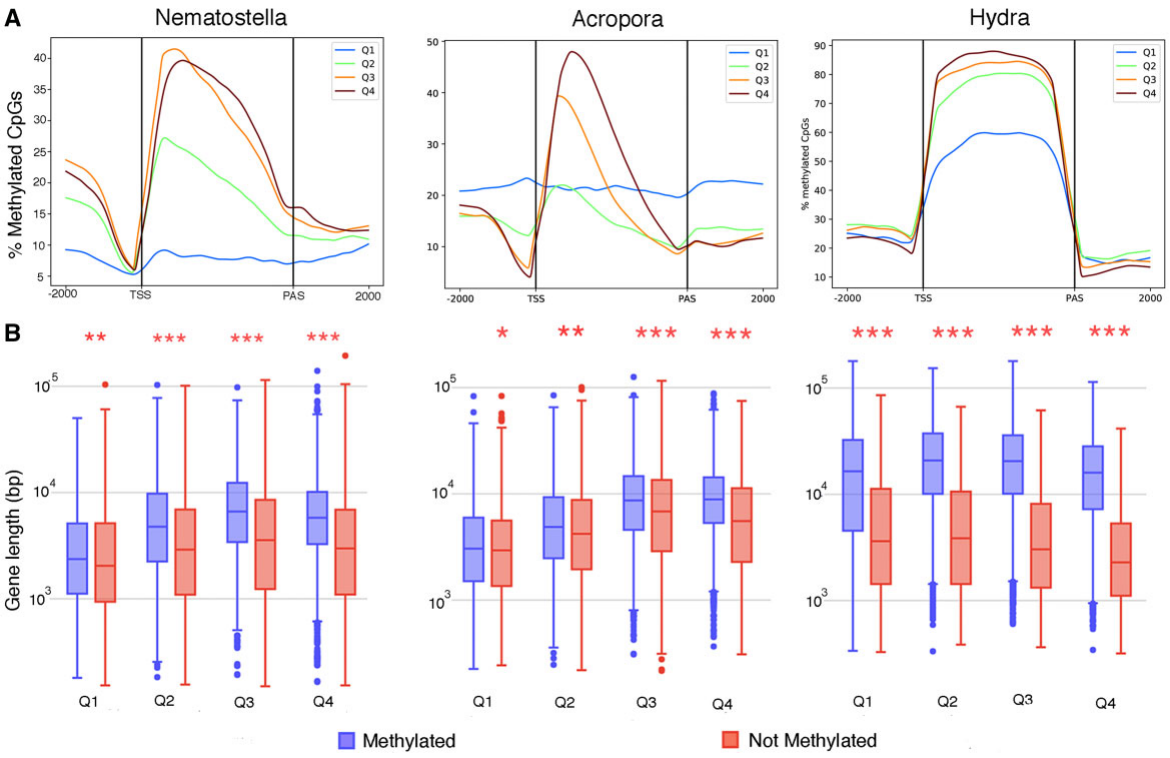
(*A*) Methylation of gene bodies is higher in genes that are more highly expressed in the three cnidarians. The percentage of CpG methylation was calculated from 2 kb upstream of the TSS (transcription start site) to 2 kb downstream of the PAS (polyadenylation site) for genes (≥2 kb in length) in the lowest (Q1) to highest (Q4) quartiles with respect to expression levels. The smoothed line shows the median for each partition, calculated by dividing each section into equal parts (*n* = 100 for gene body; and *n* = 50 for upstream and downstream) and then calculating the ML for each part. (*B*) Comparison of the lengths of methylated and unmethylated genes in different expression quartiles across the three cnidarians. The asterisks indicate *P* values from the comparison of the two distributions, as follows: *P < 0.05, **P < 0.001, ***P < 10^−16^.

Several studies have reported that gbM level increases with gene expression level in various invertebrates, including *Acropora* (Dimond and Roberts 2016), leading us to ask whether this relationship might be explained by increased transposon methylation in more highly expressed genes. To investigate this relationship, expressed genes (RPKM > 0) were grouped into four equal categories, expression quartiles; based on relative expression levels, Q4 represents the most highly expressed 25% of genes and Q1 represents the 25% of genes with the lowest expression levels. For the three cnidarians studied here, increased transcriptional activity was generally accompanied by higher mCpG levels over a region from the 5′-end to the center of the gene body before falling toward the 3′-end (fig. 4*A*). This pattern is quite different to that seen in, for example, the honeybee, where mCpGs were concentrated at the 5′-end but depleted in the middle of genes (Zemach et al. 2010). In genes expressed at relatively low levels (Q1 gene group), introns were methylated to a similar level as exons. This was in sharp contrast to highly expressed genes (Q4 gene group) where introns were more highly methylated than exons (Q4 gene group; supplementary fig. S3, Supplementary Material online). These differences were primarily due to increased intronic transposon methylation in highly expressed genes. Whereas in *Nematostella*, 34.5% of intronic transposons in all expressed genes were hypermethylated (≥70%), this was true of only 6.2% of intronic transposons in Q1 genes, and the corresponding figures for *Acropora* genes were 31.9% and 18.5%. In the case of *Hydra*, intronic transposons in all expressed genes were mostly hypermethylated (supplementary fig. S4, Supplementary Material online), as described below. Note that, for statistical purposes, only individual transposons with ≥10 covered CpGs were included in these analyses, and that individual transposons appeared to be either unmethylated or hypermethylated.

The observation that transposon methylation increased with gene expression level raised the question of whether it is a by-product of the increasing exon methylation or is independent of it. To investigate this question, length distributions were compared between unmethylated (ML ≤ 10%) and methylated (ML > 10%) genes. In each of the three cnidarians studied here, methylated genes were significantly longer than were unmethylated genes across the Q2–Q4 expression quartiles; for Q1 genes such differences were much less pronounced (as in *Nematostella* and *Acropora*) (fig. 4*B*). By contrast, the opposite trend (i.e., shorter genes were more highly methylated than were longer genes) was shown by the honeybee (Sarda et al. 2012), where the genome harbors very limited TE methylation (Lewis et al. 2020). These results suggest that, rather than being correlated with exon methylation, transposons may be independently targeted for DNA methylation in cnidarians, and that increased intronic transposon methylation is a major contributor to the increased overall ML in more highly expressed genes.

### Intragenic Hypermethylation Was Driven by Transposon Expansion in *Hydra*

Among invertebrates, the *Hydra* genome is atypical in having hypermethylated gene bodies against a mosaic background. Moreover, at approximately 900 Mb, the *Hydra* (*vulgaris*) genome is much larger than that of *Hydra viridissima* (280 Mb), the earliest diverging member of the genus (Hamada et al. 2020). The large size of the *Hydra* genome is a consequence of rapid expansion (over <87 Ma; Wong et al. 2019) of non-long terminal repeat retrotransposons, which account for over 50% of the genome and many of which are still active (Chapman et al. 2010). This combination of factors provided an opportunity to investigate relationships between DNA methylation and aspects of genome organization.

The expansion of the *Hydra* genome had little influence on the size of transcripts, but substantially increased the average intron size (supplementary table S8, Supplementary Material online). As a result, *Hydra* genes are significantly longer than those of *Nematostella* or *Acropora*. The violin plot of gene length distribution (supplementary fig. S5, Supplementary Material online) suggests a bimodal distribution for *Hydra* intron expansion with a boundary between them at approximately 5 kb. This point was chosen as an empirical boundary for defining short and long genes for further analyses.

**Fig. 5.**
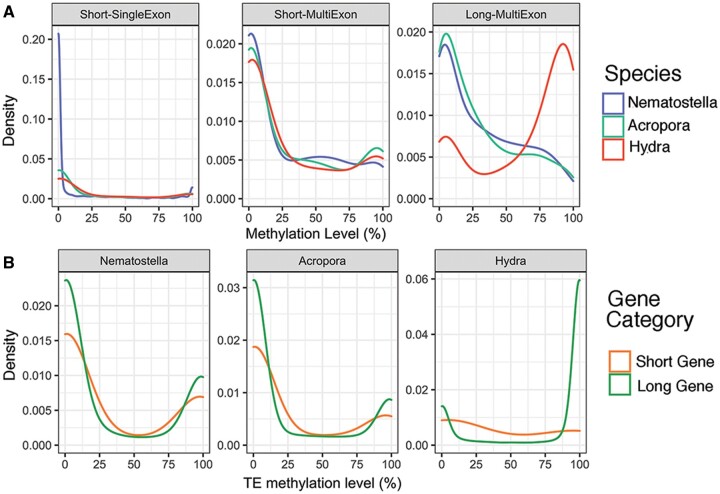
(*A*) Kernel density plot of DNA ML differences among the three cnidarians on short and long genes. Hypermethylation of long genes in *Hydra* is primarily due to methylation of intronic transposons. Although short single- (left) or multiexon (middle) genes are methylated to a similar extent in *Hydra* (red), *Nematostella* (purple), and *Acropora* (green), a much higher proportion of long multiexon genes (right panel) in *Hydra* are hypermethylated than in the other species. (*B*) Comparison of intronic transposon ML distribution located in short and long genes across the three cnidarians.

When MLs for individual short and long genes were compared across the three cnidarian species (fig. 5), most of those classified as long genes in *Hydra* were highly methylated compared with the corresponding class in *Nematostella* and *Acropora*, whereas in the case of short genes (which included both single- and multi-exon genes), DNA ML distributions were indistinguishable among the three cnidarians (fig. 5*A*). Indeed, 41% of Q1 and 71.1% of Q2–Q4 long genes were hypermethylated (ML ≥ 70%) in the *Hydra* genome. In striking contrast, only 14.2% and 13.3% of long Q2–Q4 genes in *Nematostella* and *Acropora* were hypermethylated, respectively. Therefore, the vertebrate-like level of gbM in *Hydra* is a consequence of intron expansion when gene lengths exceeded a specific threshold (e.g., 5 kb in this study).

Since transposon expansion appears to be the driving force for intron expansion, we further investigated its contribution to the high gbM in the *Hydra* genome. Consistent with the trend from gbM, approximately 75% of transposons in expressed long genes were hypermethylated (≥70%) as opposed to approximately 25% hypermethylated transposons in expressed short genes (fig. 5*B*). In comparison, transposons located in short and long genes displayed smaller differences in the *Nematostella* and *Acropora* genomes (fig. 5*B*). By providing more sites for the spreading effect of methylation (Choi and Lee 2020) to propagate from, the higher density of transposons in *Hydra* introns (>50%) compared with those of *Nematostella* and *Acropora* (∼30%) potentially explains the higher exon ML in *Hydra* than in *Acropora* and *Nematostella*.

We conclude that transposon methylation is the primary driver for the vertebrate-like gbM in the *Hydra* genome. Our results provide further support for the hypothesis that DNA methylation directly targets transposons independently of exon methylation. Moreover, that this is also a major cause of the elevated DNA MLs observed on exons and intronic nonrepetitive sequences in *Hydra* compared with *Nematostella* and *Acropora*.

### Correlation between Transposon Content, DNA Methylation, and Genome Size in Cnidarians

From the above, it is clear that variation in DNA MLs on transposons contribute significantly to different genome-wide DNA MLs in cnidarians. The observation that DNA methylation preferentially targets what are likely to be the most active transposons implies that DNA methylation plays an important role in genome defense, leading us to predict a positive correlation between genome size or transposon content and overall levels of DNA methylation in the genome.

**Fig. 6.**
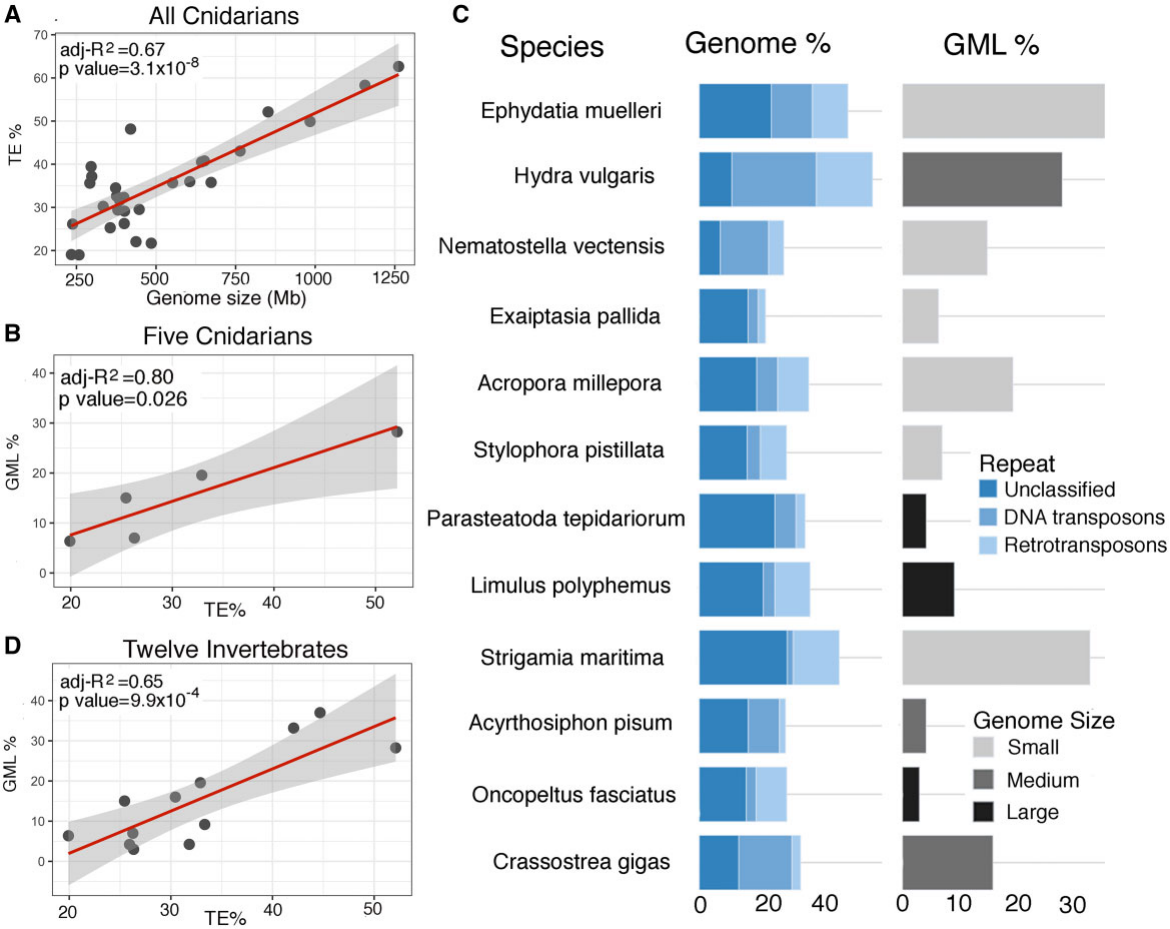
Relationships between genome size, transposon content, and genome-wide ML in Cnidaria and invertebrates more broadly. (*A*) Relationship between genome size and transposon content across the Cnidaria (adjusted *R*^2^=0.67, *P* = 3.1×10^−8^). (*B*) Correlation between transposon content and genome-wide methylation in cnidarians. A strong correlation (adjusted *R*^2^=0.80, *P* = 0.026) was observed between transposon content and the extent of methylation across the Cnidaria. (*C*) Despite extensive variation in both transposon content (18.99–62.66%) and genome size (234–1,262 Mb), strong correlation (adjusted *R*^2^=0.65, *P* = 9.9×10^−4^) was observed (*D*) between genome-wide MLs (*y* axis) and transposon content (*x* axis) across phylogenetically diverse invertebrates.

We examined the relationship between genome size and transposon content of a larger number of cnidarians, by adding data for seven additional species of Actiniaria, 15 of Scleractinia, and four of Hydrozoa (supplementary table S9, Supplementary Material online) to the three species that are the focus of this study. For these additional species, assembled sequence size was used as genome size, and transposon content was based on the percentage of the genome classified as interspersed repeats by de novo annotation. Examination of this expanded cnidarian data set, for which both genome size (234–1,262 Mb) and transposon content (18.99–62.66%) varied widely, revealed a strong correlation between genome size and transposon content (Pearson’s correlation coefficient *r* = 0.83, adj-*R*^2^ = 0.67, *P* = 3.12×10^−8^, fig. 6*A*), suggesting that transposon expansion is a major evolutionary force to increase cnidarian genome sizes.

**Figure:**
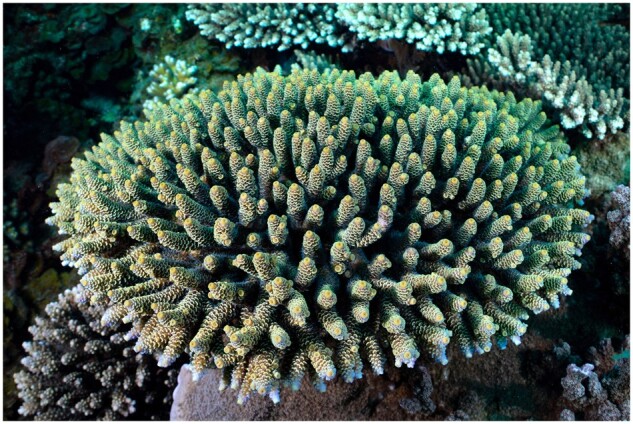
Cover image by Tom Bridge: A colony of *Acropora millepora*, one of the organisms for which methylome analyses were conducted, from the Keppel Islands on the Great Barrier Reef.

Although genome assemblies are now available for a wide range of cnidarians, methylome data are available for only two species, *Exaiptasia pallida* (sea anemone) and *Stylophora pistillata* (scleractinian coral) in addition to those presented here. Evaluation of DNA ML (which ranged from 6.37% to 28.3%) as a function of transposon content across the five cnidarian species revealed a strong positive linear relationship (Pearson’s correlation coefficient *r* = 0.92, adj-*R*^2^ = 0.80, *P* = 0.026) (fig. 6*B*), which is consistent with the hypothesis that transposon methylation is the major contributor to variations in overall DNA ML of cnidarian genomes.

Whereas Zhou et al. (2020) reported that, for vertebrates, DNA MLs are correlated with genome sizes, this was not true of the cnidarians studied here (supplementary fig. S6*A*, Supplementary Material online), although the small number of data sets (*n* = 5) available in the latter case may also be a factor. Therefore, genome size is not as good a predictor of ML as is transposon content, or relationships between these three variables are more complex (and likely heterogeneous) across the animal kingdom.

### The Relationship between DNA Methylation and Transposon Content across the Metazoa

To explore the extent to which the relationships between genome architecture and DNA methylation in cnidarians apply more generally, these analyses were expanded to other invertebrate phyla. Arthropoda is the most extensively studied lineage in terms of the availability of DNA methylation data (Lewis et al. 2020) but many species are not suitable for comparative analyses as they have incomplete DNA methylation machinery (i.e., have lost either DNMT1/3 and/or ALKB2 genes), resulting in loss of DNA ML variance (GML < 2%). For comparative purposes, arthropod species with the canonical DNA methylation repertoire and for which genome assemblies with ≤20% unclosed gaps and ≥5× BS-seq coverage were selected. Only five arthropod species met these criteria—the chelicerates *Limulus polyphemus* and *Parasteatoda tepidariorum*, the myriapod *Strigamia maritima*, and the hemimetabolous insects *Oncopeltus fasciatus* and *Acyrthosiphon pisum*. To broaden our phyletic coverage, data for the oyster *Crassostrea gigas* (Mollusca, Wang et al. 2014) and the sponge *Ephydatia muelleri* (Kenny et al. 2020) were included in these analyses; note that these are the only other nondeuterostome invertebrates (see below) with mosaic-like DNA methylation patterns (supplementary table S10, Supplementary Material online) for which data passing the criteria above were found.

Unlike the situation in cnidarians, no correlation was observed between genome size and transposon content in arthropods or in the other invertebrates included in the analyses (fig. 6*C* and supplementary table S10, Supplementary Material online). The transposon contents of the three largest arthropod genomes (>1 Gb; 26.33–33.33%) were significantly lower than those of *Strigamia maritima* (176 Mb; transposon content 42.08%) or the sponge *Ephydatia muelleri* (322 Mb; transposon content 44.68%). In the case of arthropods, consideration of divergence revealed that the larger (>1 Gb) genomes harbored a higher proportion of “middle-aged” (supplementary table S10, Supplementary Material online) transposons than did the small genomes (<500 Mb), suggesting that bursts of transposon insertions may have driven genome expansion in the deep past. Consequently, it is apparent that evolutionary forces that shaped genome architecture are likely to differ, both among arthropod lineages and other invertebrate phyla.

Despite lack of correlation between transposon content and genome size within invertebrate phyla, the positive correlation between DNA ML and transposon content was well-maintained. The seven additional invertebrate genomes exhibited larger variation in DNA MLs (from 3.00% in *Oncopeltus fasciatus* to 33.18% in *Strigamia maritima*) than did cnidarians (fig. 6*C*). Evaluating the extent of DNA methylation as a function of transposon content revealed that about 65% of the variation is accounted for by the differences in transposon content across the 12 invertebrate genomes (fig. 6*D*). It should be noted that the estimates used here are based on recognizable transposon complements, and the DNA MLs were derived from CpGs currently methylated in the genome. Therefore, present transposon burden is the single dominant factor that accounts for the enormous range of variation in genome DNA ML among invertebrate lineages that diverged before or in the early Cambrian.

## Discussion

Regardless of any involvement in regulating gene expression, it is clear that DNA methylation plays an important role in protecting cnidarian genomes against potentially harmful impacts of transposon activity. Although the consensus has been otherwise, here we present evidence that DNA methylation specifically targets intragenic and intergenic transposons, and that transposon methylation appears to be a major contributor to both global DNA MLs and to the elevated levels of gbM observed in more highly expressed genes in members of this phylum. This pattern is most obvious in the case of *Hydra*, where the size of the genome has increased as a result of bursts of transposon expansion (Wong et al. 2019). In addition, the extent of transposon methylation was strongly affected by its location (intronic or intergenic), local environment (heterochromatic/euchromatic, expression level, local transposon density), and “age,” in that young transposons located in long and highly expressed genes were those most likely to be methylated. Although the estimates of DNA MLs presented are essentially snapshots of states that have been found to differ across a species range as well as between developmental stages and tissue types, these differences are small (usually 1–5%) compared with those between species (3–37% in this study). On this basis, such variations in GML within a species do not affect our conclusions.

As young transposons are also those more likely to be active, their preferential methylation implies that repression of transposon activity (i.e., genome protection) may be the primary role of DNA methylation in cnidarians. In vertebrates, transposons remain heavily methylated beyond the point of having lost function (Mills et al. 2007; Bourque et al. 2018), but this is not the case in cnidarians, suggesting that inactive transposons have a tendency to lose DNA methylation over time. This might represent an evolutionary tradeoff, in that the overall level of DNA methylation is sufficient to suppress deleterious transposon activity while undesirable side effects of DNA methylation (Choi and Lee 2020), such as elevated mutation rate on mCpGs (Duncan and Miller 1980; Bulmer 1986) and silencing of neighboring genes (Morgan et al. 1999; Hollister and Gaut 2009; Borgognone et al. 2018) is avoided.

We suggest that the DNA methylation profile observed in cnidarians may represent an ancestral pattern that has been conserved but elaborated on in other invertebrate phyla. It has been shown that gbM is universal in metazoans where 5mC is present (Feng et al. 2010; Zemach et al. 2010), and transposon methylation has previously been reported in the oyster (Wang et al. 2014), sea urchin (Bird et al. 1979; Xu et al. 2019), and sea squirt (Keller et al. 2016). Phylogenetic reconstruction based on a broad range of arthropod methylomes implies that transposon methylation was an ancestral characteristic (Lewis et al. 2020). Transposon methylation is also evident in all three sponge species for which methylome data are available (de Mendoza et al. 2019; Kenny et al. 2020). Therefore, the gbM with transposon methylation pattern is present from the earliest diverging metazoans (sponges and cnidarians) to members of the Ecdysozoa (arthropods), Lophotrochozoa (oyster), and invertebrate deuterostomes (sea urchin, urochorate). Although mechanisms may differ, a positive correlation (between GML and transposon content) is seen in flowering plants (Niederhuth et al. 2016); transposons are also targeted by methylation in fungi (Castanera et al. 2016) and some protists (Harony and Ankri 2008).

Methylation of transposons in invertebrates presumably leads to formation of localized heterochromatin-like structures in a manner analogous to those formed after CHG methylation of transposons in plants. The presence of heterochromatic regions within highly active genes may appear paradoxical, but has clear precedents in plants (see, for example, Espinas et al. [2020]) and is likely to apply more generally given the extensive methylation of transposons typical of vertebrate genomes. A role of this kind provides a simple rationalization for the high level of conservation of the CpG methylation apparatus (MBDs, SRA proteins, histone deacetylases, heterochromatin proteins, etc.) observed across the Metazoa.

The correlation between transposon content and genome size observed in cnidarians also holds for insects with relatively small genomes, that is, those with <1 pg/N (∼1 Gb), but not for other arthropods or more widely (Canapa et al. 2015). Genome size is subject to evolutionary constraints that may differ in nature and strength between lineages; factors that limit genome size include cell size (Cavalier-Smith 2005) as well as the energetic and nutritional (particularly N and S availability in the case of corals, [Morris et al. 2019]) costs of replicating DNA.

Despite the lack of a universal relationship between genome size and transposon content, the strong positive correlation between DNA ML and transposon content is maintained beyond cnidarians to representatives of multiple invertebrate phyla. Although we suggest that this relationship reflects the ancestral invertebrate condition, deviations from that pattern are common. The invertebrate deuterostomes *Strongylocentrotus purpuratus* (sea urchin) and *Ciona intestinalis* (sea squirt, a urochordate) exhibited unusually high global DNA MLs relative to their transposon content (supplementary fig. S6*B*, Supplementary Material online), possibly reflecting the imposition of secondary (vertebrate-like) roles on the methylation system. Whereas methylation reprograming has been considered a vertebrate-specific trait, recent work (Xu et al. 2019) implies that some degree of methylation reprogramming occurs during embryonic development in both sea urchin and urochordate. The fact that the genomes of these organisms have elevated GML relative to their transposon content might therefore be a consequence of the emergence of developmental reprogramming systems in invertebrate deuterostomes. Likewise, the imposition of secondary roles on DNA methylation might likewise explain the vertebrate-like methylomes of the sponges *Amphimedon* and *Sycon* (de Mendoza et al. 2019). However, as such roles remain elusive, these species were excluded from our analyses. Loss of all or some of the DNA methylation repertoire also results in complex relationships between transposon content and genome size as well as between GML and transposon content. Such losses have occurred in a number of lineages; the case of *Caenorhabditis* was mentioned above, and additional cases include *Oikopleura dioica*, Placozoa, and myxozoan cnidarians (Albalat et al. 2012; de Mendoza et al. 2019; Kyger et al. 2021).

DNA methylation carries significant evolutionary costs, which include not only the high mutability of mCpG (an order of magnitude higher than for CpG), but also the production of the toxic cytosine derivative 3-methylcytosine (3-MeC) by both the maintenance methylase DNMT1 and the de novo methylase DNMT3 (Rošić et al. 2018). The dealkylation of 3-MeC to cytosine is carried out by ALK2 but the presence of 3-MeC may still cause DNA damage (double-stranded DNA breakage) by stalling DNA polymerase (Rošić et al. 2018). Maintaining the DNA-methylation system therefore requires conservation not only of the DNA methylases, TET, and the reading machinery, but also of the DNA dealkylation enzyme ALK2 and the double-strand (DS) break repair system. Note that RAD18 and the BRCA complex, key components of the DS break repair system, have coevolved with the DNMTs (Rošić et al. 2018). At least some functions of DNA methylation are redundant; recombination and gene conversion can remove TEs (Huttley et al. 1995), the Piwi system also acts to constrain transposon activity in the germ line and, on evolutionary time scales, some aspects of the histone code may be functionally equivalent to DNA methylation states (Nanty et al. 2011). On these bases, it is hardly surprising that a number of organisms have either completely lost the capacity to carry out DNA methylation, or have lost some components of the methylation machinery (close inspection of data in Lewis et al. [2020] implies that this is the case in holometabolous insects).

It is worth noting that the analyses presented here are based on sequencing bisulfite-modified DNA and thus provide real-time data. Therefore, in invertebrates, DNA methylation responds to the current transposon burden in the genomes, irrespective of how transposon content, genome size, and DNA methylation varied in the past. Thus, our study strongly supports Bestor’s genome defense hypothesis in that DNA methylation is essentially a host immune response to transposon invasion, and that this applies to invertebrates as well as vertebrates. A role for methylation in suppressing transposon activity provides a rationalization for not only the high degree of conservation of the transcriptional silencing machinery from sponges to mammals, but also the wide range of overall GML observed—as it is difficult to account for GML varying >10-fold if methylation served only regulatory roles.

## Materials and Methods

### Biological Material

#### Nematostella

*Nematostella* animals in laboratory culture were F1 offspring of CH2XCH6 individuals collected from the Rhode River, MD. They were kept under constant, artificial conditions without substrate or light in plastic boxes filled with *Nematostella* medium (NM), which was adjusted to 16‰ salinity with Red Sea Salt and Millipore H_2_O. Polyps were fed twice per week with first instar nauplius larvae of *Artemia salina* as prey (Ocean Nutrition Micro *Artemia* Cysts 430–500 gr, Coralsands, Wiesbaden, Germany) and washed once per week with media preincubated at the culture temperature. A single female polyp was selected for DNA extraction.

#### Acropora

*Acropora millepora* planulae were collected at Orpheus Island Research Station (18.61°S, 146.49°E), Australia, under GBRMPA permit G33232.1 during the mass coral spawning event in 2010, washed twice with Millipore-filtered (20 µm) sea water, and then frozen in liquid nitrogen and stored at −80 °C until needed.

#### Hydra

Genomic DNA was isolated from transgenic lines of *Hydra* expressing eGFP in the ectodermal or endodermal epithelial cell lineages (Wittlieb et al. 2006; Khalturin et al. 2007), or in the interstitial stem cells (Boehm et al. 2012; Hemmrich et al. 2012). GFP-labeled cells (0.6×10^6^, 1.9×10^6^, and 0.7×10^6^ cells ectodermal, endodermal, or interstitial stem cells, respectively) were harvested from transgenic polyps using fluorescence-activated cell sorting (FACS) as previously described (Hemmrich et al. 2012). After the cell collection a small aliquot was reanalyzed to verify the purity of sorted fractions, which in all cases exceeded 95%. Immediately after FACS isolation, sorted cells were centrifuged at 1,000 U/min for 5 min, the supernatant removed, and the resulting pellet dissolved directly in the lysis (AL) Buffer from the DNeasy Blood & Tissue Kit (Qiagen). Further processing of total DNA followed the manufacturer’s instructions. Pure DNA was eluted from the cartridges with Tris Buffer (10 mM, pH 9.0). DNA concentrations were measured by absorption at 260 nm using the Nanodrop (ThermoFisher).

### DNA Extraction, Library Construction, and BS-Seq

#### Nematostella

The polyp was starved for 3 days before sacrifice, washed two times with 2 ml aliquots of autoclaved MilliQ water, snap-frozen in liquid N_2_ without liquid, and stored at −80 °C until extraction. Genomic DNA was extracted from the adult body column only with the AllPrep DNA/RNA/miRNA Universal Kit (Qiagen, Hilden, Germany), as described in the manufacturer’s protocol. Elution of RNA and DNA was done in 20 and 50 µl, respectively, and the eluates stored at −80 °C until sequencing. DNA concentrations were measured through electrophoresis by loading 5 µl of each sample on a 1% agarose gel and by spectrophotometry on a Nanodrop 3300 (Thermo Fisher Scientific).

DNA samples were processed with the NEBNext Enzymatic Methyl-seq Kit (EM-Seq) (New England Biolabs, Ipswich) according to the “manufacturers large insert size” protocol and sequenced on the NovaSeq 6000, SP Flowcell 2×150 bp (Illumina, San Diego). NGS sequencing were carried out at the Competence Centre for Genomic Analysis (Kiel).

#### Hydra and Acropora

High-molecular weight genomic DNA (>50 kb) was prepared as described by Blin and Stafford (1976). BS-Seq was carried out at BGI (Shenzhen, China).

### Reference Genome Data Used in the Study

Genome assemblies for *Porites lutea, Galaxia fascicularis, Fungia* spp., and *Goniastrea aspera* were obtained from reef genomics repository (http://refuge2020.reefgenomics.org, last accessed January 29, 2022). The genome assembly for *Heliofungia actiniformis* was kindly provided by Dr Ira Cooke (James Cook University, Australia), and are accessible via an Apollo web browser interface at https://coral.genome.edu.au/ (last accessed January 29, 2022). The sponge, *Ephydatia muelleri*, genome data (Kenny et al. 2020) were downloaded from https://bitbucket.org/EphydatiaGenome/ephydatiagenome (last accessed January 29, 2022). All the other invertebrate genome data were downloaded from NCBI assembly or ENSEMBL metazoan databases. The accession numbers are listed in supplementary table S1, Supplementary Material online.

### Analysis of BS-Seq Data

BS-seq reads were trimmed of sequencing adapters and low-quality bases (score < 20) using TrimGalore v0.6.0 (https://github.com/FelixKrueger/TrimGalore, last accessed January 29, 2022). To avoid methylation bias (M-bias), 9 bp from both 5′- and 3′-ends were further trimmed. Only paired end reads with read length greater than 30 bp were retained. BisMark v0.20.0 (Krueger and Andrews 2011) was applied to map reads to the reference genomes. Reads that mapped to multiple locations, and clonal reads were removed. The perl script bismark2report provided by BisMark was used to generate the methylation report which showed that less than 1% methyl-cytosines were detected in CHG or CHH (H = A, G, or T) context. Therefore, we used 1% as the error rate arising from incomplete bisulfite conversion and sequencing errors. To call methylated sites, we performed a binomial test on each cytosine given the number of methylated calls, sequencing coverage (≥2), and the probability of success equal to the error rate. Multiple test corrections (Benjamini–Hochberg method) were conducted on CpG, CHG, and CHH contexts, respectively, and methylated cytosines were determined by the corrected *P* value threshold of 0.05.

### Analyses of RNA-Seq Data

RNA-seq reads from the same biological samples as BS-seq were downloaded from the Gene Expression Omnibus (GEO) or Short Read Archive (SRA) database. The accession numbers for *Nematostella, Acropora*, and *Hydra* are GSE168938, SRX5072320, and SRX019488, respectively. For *Nematostella* and *Acropora* reads (Illumina pair-end reads), Trimmomatic v.0.38 (Bolger et al. 2014) was applied to remove adaptors and low-quality bases whose quality scores were less than 20. Reads shorter than 50 bp were removed, and only paired-end reads were retained. Trimmed reads were mapped to their respective genomes using the splice-aware aligner hisat2 v2.1.0 (Kim et al. 2015) with strandedness option where appropriate and default parameters. For *Hydra* reads (454 sequencing reads), the tag sequences were detected and cleaned by TagCleaner (https://sourceforge.net/projects/tagcleaner/, last accessed January 29, 2022). BBduk v38.37 (BBMap, Bushnell B., sourceforge.net/projects/bbmap/, last accessed January 29, 2022) was applied to trim 40 bp from 5′-end, bases after position 150, and low-quality (<20) bases. Trimmed reads shorter than 50 bp were further discarded. Magicblast v1.4.0 (https://bmcbioinformatics.biomedcentral.com/articles/10.1186/s12859-019-2996-x) was applied to align trimmed reads to the *Hydra* genome with default settings. Read fragments that were aligned to the annotated genes were counted using the FeatureCounts program (Liao et al. 2014) with default parameters. Gene expression level was estimated from reads (or fragments if paired-end) per kilobase per million reads (RPKM or FPKM) (Mortazavi et al. 2008).

### De Novo Repeat Annotation

Repetitive elements were annotated for all the genomes used in this study. First, a de novo repeat library was generated with Repeat-Modeler (Version 1.0.11, http://www.repeatmasker.org/RepeatModeler/, last accessed January 29, 2022) with default parameters. This library was combined with the RepBase database (https://www.girinst.org/repbase/, last accessed January 29, 2022) and used as input for RepeatMasker (version 4.0.8, http://www.repeatmasker.org, last accessed January 29, 2022) to identify repeat categories and locations. For each interspersed repeat reported by RepeatMasker, the Kimura distance value was extracted from the alignment file and interpreted as the divergence to the consensus sequence of the corresponding transposon family. 

## Supplementary Material

Supplementary data are available at *Molecular Biology and Evolution* online.

## Supplementary Material

msac018_Supplementary_Data

## References

[msac018-B1] Albalat R , Martí-Solans J, Cañestro C. 2012. DNA methylation in amphioxus: from ancestral functions to new roles in vertebrates. Brief Funct Genomics. 11(2):142–155. 22389042 10.1093/bfgp/els009

[msac018-B2] Ball EE , Hayward DC, Bridge TCL, Miller DJ. 2021. *Acropora* – the most studied coral genus. In: Boutet A, Schierwater B, editors. Handbook of marine model organisms in experimental biology – established and emerging. Boca Raton (FL): CRC Press. p. 173–193.

[msac018-B3] Bestor TH. 1990. DNA methylation: evolution of a bacterial immune function into a regulator of gene expression and genome structure in higher eukaryotes. Phil Trans R Soc Lond B. 326(1235):179–187. 1968655 10.1098/rstb.1990.0002

[msac018-B4] Bestor T , Laudano A, Mattaliano R, Ingram V. 1988. Cloning and sequencing of a cDNA encoding DNA methyltransferase of mouse cells: the carboxyl-terminal domain of the mammalian enzymes is related to bacterial restriction methyltransferases. J Mol Biol. 203(4):971–983. 3210246 10.1016/0022-2836(88)90122-2

[msac018-B5] Bestor TH , Tycko B. 1996. Creation of genomic methylation patterns. Nat Genet. 12(4):363–367. 8630488 10.1038/ng0496-363

[msac018-B6] Bestor TH , Edwards JR, Boulard M. 2015. Notes on the role of dynamic DNA methylation in mammalian development. Proc Natl Acad Sci U S A. 112(22):6796–6799. 25368180 10.1073/pnas.1415301111 PMC4460495

[msac018-B7] Bewick AJ , Vogel KJ, Moore AJ, Schmitz RJ. 2017. Evolution of DNA methylation across insects. Mol Biol Evol. 34(3):654–665. 28025279 10.1093/molbev/msw264 PMC5400375

[msac018-B8] Bird A , Taggart MH, Smith BA. 1979. Methylated and unmethylated compartments in the sea urchin genome. Cell 17(4):889–901. 487434 10.1016/0092-8674(79)90329-5

[msac018-B9] Bird A , Tate P, Nan X, Campoy J, Meehan R, Cross S, Tweedie S, Charlton J, Macleod D. 1995. Studies of DNA methylation in animals. J Cell Sci Suppl. 19:37–39. 8655645 10.1242/jcs.1995.supplement_19.5

[msac018-B10] Blin N , Stafford DW. 1976. A general method for isolation of high molecular weight DNA from eukaryotes. Nucleic Acids Res. 3(9):2303–2308. 987581 10.1093/nar/3.9.2303 PMC343085

[msac018-B11] Boehm A-M , Khalturin K, Anton-Erxleben F, Hemmrich G, Klostermeier UC, Lopez-Quintero JA, Oberg H-H, Puchert M, Rosenstiel P, Wittlieb J, et al 2012. FoxO is a critical regulator of stem cell maintenance and immortality in Hydra. Proc Natl Acad Sci U S A. 109(48):19697–19702. 23150562 10.1073/pnas.1209714109 PMC3511741

[msac018-B12] Bolger AM , Lohse M, Usadel B. 2014. Trimmomatic: a flexible trimmer for Illumina sequence data. Bioinformatics 30(15):2114–2120. 24695404 10.1093/bioinformatics/btu170 PMC4103590

[msac018-B13] Borgognone A , Castanera R, Morselli M, López-Varas L, Rubbi L, Pisabarro AG, Pellegrini M, Ramírez L. 2018. Transposon-associated epigenetic silencing during *Pleurotus ostreatus* life cycle. DNA Res. 25(5):451–464. 29893819 10.1093/dnares/dsy016 PMC6191308

[msac018-B14] Bourque G , Burns KH, Gehring M, Gorbunova V, Seluanov A, Hammell M, Imbeault M, Izsvák Z, Levin HL, Macfarlan TS, et al 2018. Ten things you should know about transposable elements. Genome Biol. 19(1):1–12. 30454069 10.1186/s13059-018-1577-z PMC6240941

[msac018-B15] Bulmer M. 1986. Neighboring base effects on substitution rates in pseudogenes. Mol Biol Evol. 3(4):322–329. 3444408 10.1093/oxfordjournals.molbev.a040401

[msac018-B16] Canapa A , Barucca M, Biscotti MA, Forconi M, Olmo E. 2015. Transposons, genome size, and evolutionary insights in animals. Cytogenet Genome Res. 147(4):217–239. 26967166 10.1159/000444429

[msac018-B17] Castanera R , Lopez-Varas L, Borgognone A, LaButti K, Lapidus A, Schmutz J, Grimwood J, Perez G, Pisabarro AG, Grigoriev IV, et al 2016. Transposable elements versus the fungal genome: impact on whole-genome architecture and transcriptional profiles. PLoS Genet. 12(6):e1006108. 27294409 10.1371/journal.pgen.1006108 PMC4905642

[msac018-B18] Cavalier-Smith T. 2005. Economy, speed and size matter: evolutionary forces driving nuclear genome miniaturization and expansion. Ann Bot. 95(1):147–175. 15596464 10.1093/aob/mci010 PMC4246715

[msac018-B19] Chapman JA , Kirkness EF, Simakov O, Hampson SE, Mitros T, Weinmaier T, Rattei T, Balasubramanian PG, Borman J, Busam D, et al 2010. The dynamic genome of Hydra. Nature 464(7288):592–596. 20228792 10.1038/nature08830 PMC4479502

[msac018-B20] Choi JY , Lee Y-CG. 2020. Double-edged sword: the evolutionary consequences of the epigenetic silencing of transposable elements. PLoS Genet. 16(7):e1008872. 32673310 10.1371/journal.pgen.1008872 PMC7365398

[msac018-B21] Compere SJ , Palmiter RD. 1981. DNA methylation controls the inducibility of the mouse metallothionein-I gene in lymphoid cells. Cell 25(1):233–240. 6168387 10.1016/0092-8674(81)90248-8

[msac018-B22] Cosby RL , Chang N-C, Feschotte C. 2019. Host-transposon interactions: conflict, cooperation and cooption. Genes Dev. 33(17–18):1098–1116. 31481535 10.1101/gad.327312.119 PMC6719617

[msac018-B23] de Mendoza A , Hatleberg WL, Pang K, Leininger S, Bogdanovic O, Pflueger J, Buckberry S, Technau U, Hejnol A, Adamska M, et al 2019. Convergent evolution of a vertebrate-like methylome in a marine sponge. Nat Ecol Evol. 3(10):1464–1473. 31558833 10.1038/s41559-019-0983-2 PMC6783312

[msac018-B24] Dimond JL , Roberts SB. 2016. Germline DNA methylation in reef corals: patterns and potential roles in response to environmental change. Mol Ecol. 25(8):1895–1904. 26454152 10.1111/mec.13414

[msac018-B25] Dimond JL , Roberts SB. 2020. Convergence of DNA methylation profiles of the reef coral *Porites astreoides* in a novel environment. Front Mar Sci. 6:792.

[msac018-B26] Dixon G , Liao Y, Bay LK, Matz MV. 2018. Role of gene body methylation in acclimatization and adaptation in a basal metazoan. Proc Natl Acad Sci U S A. 115(52):13342–13346. 30530646 10.1073/pnas.1813749115 PMC6310852

[msac018-B27] Dixon G , Matz M. 2021. Benchmarking DNA methylation assays in a reef‐building coral. Mol Ecol Resour. 21(2):464–477. 33058551 10.1111/1755-0998.13282

[msac018-B28] Dixon GB , Bay LK, Matz MV. 2014. Bimodal signatures of germline methylation are linked with gene expression plasticity in the coral *Acropora millepora*. BMC Genomics 15(1):1–11. 25511458 10.1186/1471-2164-15-1109 PMC4378018

[msac018-B29] Dixon GB , Bay LK, Matz MV. 2016. Evolutionary consequences of DNA methylation in a basal metazoan. Mol Biol Evol. 33(9):2285–2293. 27189563 10.1093/molbev/msw100 PMC4989105

[msac018-B30] Duncan BK , Miller JH. 1980. Mutagenic deamination of cytosine residues in DNA. Nature 287(5782):560–561. 6999365 10.1038/287560a0

[msac018-B31] Durante MK , Baums IB, Williams DE, Vohsen S, Kemp DW. 2019. What drives phenotypic divergence among coral clonemates of *Acropora palmata*? Mol Ecol. 28(13):3208–3224. 31282031 10.1111/mec.15140 PMC6852117

[msac018-B32] Espinas NA , Tu LN, Furci L, Shimajiri Y, Harukawa Y, Miura S, Takuno S, Saze H. 2020. Transcriptional regulation of genes bearing intronic heterochromatin in the rice genome. PLoS Genet. 16(3):e1008637. 32187179 10.1371/journal.pgen.1008637 PMC7145194

[msac018-B33] Feng S , Cokus SJ, Zhang X, Chen P-Y, Bostick M, Goll MG, Hetzel J, Jain J, Strauss SH, Halpern ME, et al 2010. Conservation and divergence of methylation patterning in plants and animals. Proc Natl Acad Sci U S A. 107(19):8689–8694. 20395551 10.1073/pnas.1002720107 PMC2889301

[msac018-B34] Giordano J , Ge Y, Gelfand Y, Abrusán G, Benson G, Warburton PE. 2007. Evolutionary history of mammalian transposons determined by genome-wide defragmentation. PLoS Comput Biol. 3(7):e137. 17630829 10.1371/journal.pcbi.0030137 PMC1914374

[msac018-B35] Gregory TR. 2010. Animal genome size database. Available from: http://www.genome size.com/.

[msac018-B36] Grunau C , Renault E, Rosenthal A, Roizes G. 2001. MethDB—a public database for DNA methylation data. Nucleic Acids Res. 29(1):270–274. 11125109 10.1093/nar/29.1.270 PMC29842

[msac018-B37] Hamada M , Satoh N, Khalturin K. 2020. A Reference Genome from the symbiotic hydrozoan, *Hydra viridissima*. G3 (Bethesda) 10(11):3883–3895. 32900905 10.1534/g3.120.401411 PMC7642931

[msac018-B38] Harony H , Ankri S. 2008. What do unicellular organisms teach us about DNA methylation? Trends Parasitol. 24(5):205–209. 18403268 10.1016/j.pt.2008.02.002

[msac018-B39] Hemmrich G , Khalturin K, Boehm AM, Puchert M, Anton-Erxleben F, Wittlieb J, Klostermeier UC, Rosenstiel P, Oberg H-H, Domazet-Lošo T, et al 2012. Molecular signatures of the three stem cell lineages in *Hydra* and the emergence of stem cell function at the base of multicellularity. Mol Biol Evol. 29(11):3267–3280. 22595987 10.1093/molbev/mss134

[msac018-B40] Holliday R , Pugh JE. 1975. DNA modification mechanisms and gene activity during development. Science 187(4173):226–232. 1111098

[msac018-B41] Hollister JD , Gaut BS. 2009. Epigenetic silencing of transposable elements: a trade-off between reduced transposition and deleterious effects on neighboring gene expression. Genome Res. 19(8):1419–1428. 19478138 10.1101/gr.091678.109 PMC2720190

[msac018-B42] Huttley GA , MacRae AF, Clegg MT. 1995. Molecular evolution of the Ac/Ds transposable element family in pearl millet and other grasses. Genetics 139(3):1411–1419. 7768448 10.1093/genetics/139.3.1411 PMC1206466

[msac018-B43] Keller TE , Han P, Yi SV. 2016. Evolutionary transition of promoter and gene body DNA methylation across invertebrate–vertebrate boundary. Mol Biol Evol. 33(4):1019–1028. 26715626 10.1093/molbev/msv345 PMC4776710

[msac018-B44] Kenny NJ , Francis WR, Rivera-Vicéns RE, Juravel K, de Mendoza A, Díez-Vives C, Lister R, Bezares-Calderón LA, Grombacher L, Roller M, et al 2020. Tracing animal genomic evolution with the chromosomal-level assembly of the freshwater sponge *Ephydatia muelleri*. Nat Commun. 11(1):1–11. 32719321 10.1038/s41467-020-17397-w PMC7385117

[msac018-B45] Khalturin K , Anton-Erxleben F, Milde S, Plötz C, Wittlieb J, Hemmrich G, Bosch TCG. 2007. Transgenic stem cells in Hydra reveal an early evolutionary origin for key elements controlling self-renewal and differentiation. Dev Biol. 309(1):32–44. 17659272 10.1016/j.ydbio.2007.06.013

[msac018-B46] Kim D , Langmead B, Salzberg SL. 2015. HISAT: a fast spliced aligner with low memory requirements. Nat Methods. 12(4):357–360. 25751142 10.1038/nmeth.3317 PMC4655817

[msac018-B47] Krueger F , Andrews SR. 2011. Bismark: a flexible aligner and methylation caller for bisulfite-Seq applications. Bioinformatics 27(11):1571–1572. 21493656 10.1093/bioinformatics/btr167 PMC3102221

[msac018-B48] Kyger R , Luzuriaga-Neira A, Layman T, Milkewitz Sandberg TO, Singh D, Huchon D, Peri S, Atkinson SD, Bartholomew JL, Yi SV, et al 2021. Myxosporea (Myxozoa, Cnidaria) lack DNA cytosine methylation. Mol Biol Evol. 38(2):393–404. 32898240 10.1093/molbev/msaa214 PMC7826176

[msac018-B49] Lechner M , Marz M, Ihling C, Sinz A, Stadler PF, Krauss V. 2013. The correlation of genome size and DNA methylation rate in metazoans. Theory Biosci. 132(1):47–60. 23132463 10.1007/s12064-012-0167-y

[msac018-B50] Lewis SH , Ross L, Bain SA, Pahita E, Smith SA, Cordaux R, Miska EA, Lenhard B, Jiggins FM, Sarkies P. 2020. Widespread conservation and lineage-specific diversification of genome-wide DNA methylation patterns across arthropods. PLoS Genet. 16(6):e1008864. 32584820 10.1371/journal.pgen.1008864 PMC7343188

[msac018-B51] Li Y , Liew YJ, Cui G, Cziesielski MJ, Zahran N, Michell CT, Voolstra CR, Aranda M. 2018. DNA methylation regulates transcriptional homeostasis of algal endosymbiosis in the coral model *Aiptasia*. Sci Adv. 4(8):eaat2142. 30116782 10.1126/sciadv.aat2142 PMC6093633

[msac018-B52] Liao Y , Smyth GK, Shi W. 2014. featureCounts: an efficient general purpose program for assigning sequence reads to genomic features. Bioinformatics 30(7):923–930. 24227677 10.1093/bioinformatics/btt656

[msac018-B53] Lieberman MW , Beach LR, Palmiter RD. 1983. Ultraviolet radiation-induced metallothionein-I gene activation is associated with extensive DNA demethylation. Cell 35(1):207–214. 6627395 10.1016/0092-8674(83)90223-4

[msac018-B54] Liew YJ , Zoccola D, Li Y, Tambutté E, Venn AA, Michell CT, Cui G, Deutekom ES, Kaandorp JA, Voolstra CR, et al 2018. Epigenome-associated phenotypic acclimatization to ocean acidification in a reef-building coral. Sci Adv. 4(6):eaar8028. 29881778 10.1126/sciadv.aar8028 PMC5990304

[msac018-B55] Mariani CJ , Vasanthakumar A, Madzo J, Yesilkanal A, Bhagat T, Yu Y, Bhattacharyya S, Wenger RH, Cohn SL, Nanduri J, et al 2014. TET1-mediated hydroxymethylation facilitates hypoxic gene induction in neuroblastoma. Cell Rep. 7(5):1343–1352. 24835990 10.1016/j.celrep.2014.04.040 PMC4516227

[msac018-B56] Matzke MA , Primig M, Trnovsky J, Matzke AJM. 1989. Reversible methylation and inactivation of marker genes in sequentially transformed tobacco plants. EMBO J. 8(3):643–649. 16453872 10.1002/j.1460-2075.1989.tb03421.x PMC400855

[msac018-B57] Mills RE , Bennett EA, Iskow RC, Devine SE. 2007. Which transposable elements are active in the human genome? Trends Genet. 23(4):183–191. 17331616 10.1016/j.tig.2007.02.006

[msac018-B58] Morgan HD , Sutherland HG, Martin DI, Whitelaw E. 1999. Epigenetic inheritance at the agouti locus in the mouse. Nat Genet. 23(3):314–318. 10545949 10.1038/15490

[msac018-B59] Morris L , Voolstra CR, Quigley KM, Bourne DG, Bay LK. 2019. Nutrient availability and metabolism affect the stability of coral-Symbiodiniaceae symbioses. Trends Microbiol. 27(8):678–689. 30987816 10.1016/j.tim.2019.03.004

[msac018-B60] Mortazavi A , Williams BA, McCue K, Schaeffer L, Wold B. 2008. Mapping and quantifying mammalian transcriptomes by RNA-Seq. Nat Methods. 5(7):621–628. 18516045 10.1038/nmeth.1226 PMC13303166

[msac018-B61] Nanty L , Carbajosa G, Heap GA, Ratnieks F, van Heel DA, Down TA, Rakyan VK. 2011. Comparative methylomics reveals gene-body H3K36me3 in *Drosophila* predicts DNA methylation and CpG landscapes in other invertebrates. Genome Res. 21(11):1841–1850. 21940836 10.1101/gr.121640.111 PMC3205569

[msac018-B62] Niederhuth CE , Bewick AJ, Ji L, Alabady MS, Kim KD, Li Q, Rohr NA, Rambani A, Burke JM, Udall JA, et al 2016. Widespread natural variation of DNA methylation within angiosperms. Genome Biol. 17(1):194. 27671052 10.1186/s13059-016-1059-0 PMC5037628

[msac018-B63] Putnam HM , Davidson JM, Gates RD. 2016. Ocean acidification influences host DNA methylation and phenotypic plasticity in environmentally susceptible corals. Evol Appl. 9(9):1165–1178. 27695524 10.1111/eva.12408 PMC5039329

[msac018-B64] Quesneville H. 2020. Twenty years of transposable element analysis in the *Arabidopsis thaliana* genome. Mobile DNA. 11(1):1–13. 32742313 10.1186/s13100-020-00223-x PMC7385966

[msac018-B65] Regev A , Lamb MJ, Jablonka E. 1998. The role of DNA methylation in invertebrates: developmental regulation or genome defense? Mol Biol Evol. 15(7):880–891.

[msac018-B66] Roberts SB , Gavery MR. 2012. Is there a relationship between DNA methylation and phenotypic plasticity in invertebrates? Front Physiol. 2:116. 22232607 10.3389/fphys.2011.00116 PMC3249382

[msac018-B67] Rodríguez-Casariego JA , Mercado-Molina AE, Garcia-Souto D, Ortiz-Rivera IM, Lopes C, Baums IB, Sabat AM, Eirin-Lopez JM. 2020. Genome-Wide DNA methylation analysis reveals a conserved epigenetic response to seasonal environmental variation in the staghorn coral *Acropora cervicornis*. Front Mar Sci. 7:822.

[msac018-B68] Rošić S , Amouroux R, Requena CE, Gomes A, Emperle M, Beltran T, Rane JK, Linnett S, Selkirk ME, Schiffer PH, et al 2018. Evolutionary analysis indicates that DNA alkylation damage is a byproduct of cytosine DNA methyltransferase activity. Nat Genet. 50(3):452–459. 29459678 10.1038/s41588-018-0061-8 PMC5865749

[msac018-B69] Sarda S , Zeng J, Hunt BG, Yi SV. 2012. The evolution of invertebrate gene body methylation. Mol Biol Evol. 29(8):1907–1916. 22328716 10.1093/molbev/mss062

[msac018-B70] Tweedie S , Ng HH, Barlow AL, Turner BM, Hendrich B, Bird A. 1999. Vestiges of a DNA methylation system in *Drosophila melanogaster*? Nat Genet. 23(4):389–390. 10581020 10.1038/70490

[msac018-B71] Wang X , Li Q, Lian J, Li L, Jin L, Cai H, Xu F, Qi H, Zhang L, Wu F, et al 2014. Genome-wide and single-base resolution DNA methylomes of the Pacific oyster *Crassostrea gigas* provide insight into the evolution of invertebrate CpG methylation. BMC Genomics 15(1):1–12. 25514978 10.1186/1471-2164-15-1119 PMC4378273

[msac018-B72] Wang Y , Jorda M, Jones PL, Maleszka R, Ling X, Robertson HM, Mizzen CA, Peinado MA, Robinson GE. 2006. Functional CpG methylation system in a social insect. Science 314(5799):645–647. 17068262 10.1126/science.1135213

[msac018-B73] Wittlieb J , Khalturin K, Lohmann JU, Anton-Erxleben F, Bosch TCG. 2006. Transgenic Hydra allow in vivo tracking of individual stem cells during morphogenesis. Proc Natl Acad Sci U S A. 103(16):6208–6211. 16556723 10.1073/pnas.0510163103 PMC1458856

[msac018-B74] Wong WY , Simakov O, Bridge DM, Cartwright P, Bellantuono AJ, Kuhn A, Holstein TW, David CN, Steele RE, Martínez DE. 2019. Expansion of a single transposable element family is associated with genome-size increase and radiation in the genus *Hydra*. Proc Natl Acad Sci U S A. 116(46):22915–22917. 31659034 10.1073/pnas.1910106116 PMC6859323

[msac018-B75] Xu X , Li G, Li C, Zhang J, Wang Q, Simmons DK, Chen X, Wijesena N, Zhu W, Wang Z, et al 2019. Evolutionary transition between invertebrates and vertebrates via methylation reprogramming in embryogenesis. Natl Sci Rev. 6(5):993–1003. 34691960 10.1093/nsr/nwz064 PMC8291442

[msac018-B76] Yoder JA , Walsh CP, Bestor TH. 1997. Cytosine methylation and the ecology of intragenomic parasites. Trends Genet. 13(8):335–340. 9260521 10.1016/s0168-9525(97)01181-5

[msac018-B77] Zemach A , McDaniel IE, Silva P, Zilberman D. 2010. Genome-wide evolutionary analysis of eukaryotic DNA methylation. Science 328(5980):916–919. 20395474 10.1126/science.1186366

[msac018-B78] Zhou W , Liang G, Molloy PL, Jones PA. 2020. DNA methylation enables transposable element-driven genome expansion. Proc Natl Acad Sci U S A. 117(32):19359–19366. 32719115 10.1073/pnas.1921719117 PMC7431005

