# Supplementary material for: The Role of DNA Methylation in Genome Defense in Cnidaria and Other Invertebrates
Source: Mol Biol Evol. 2022 Jan 27;39(2):msac018. doi: 10.1093/molbev/msac018 (PMC8857917; doi:10.1093/molbev/msac018)
Supplement: msac018_Supplementary_Data [file msac018_supplementary_data.zip › Ying_Supplementary_figures_final.pdf]

Supplementary Figures

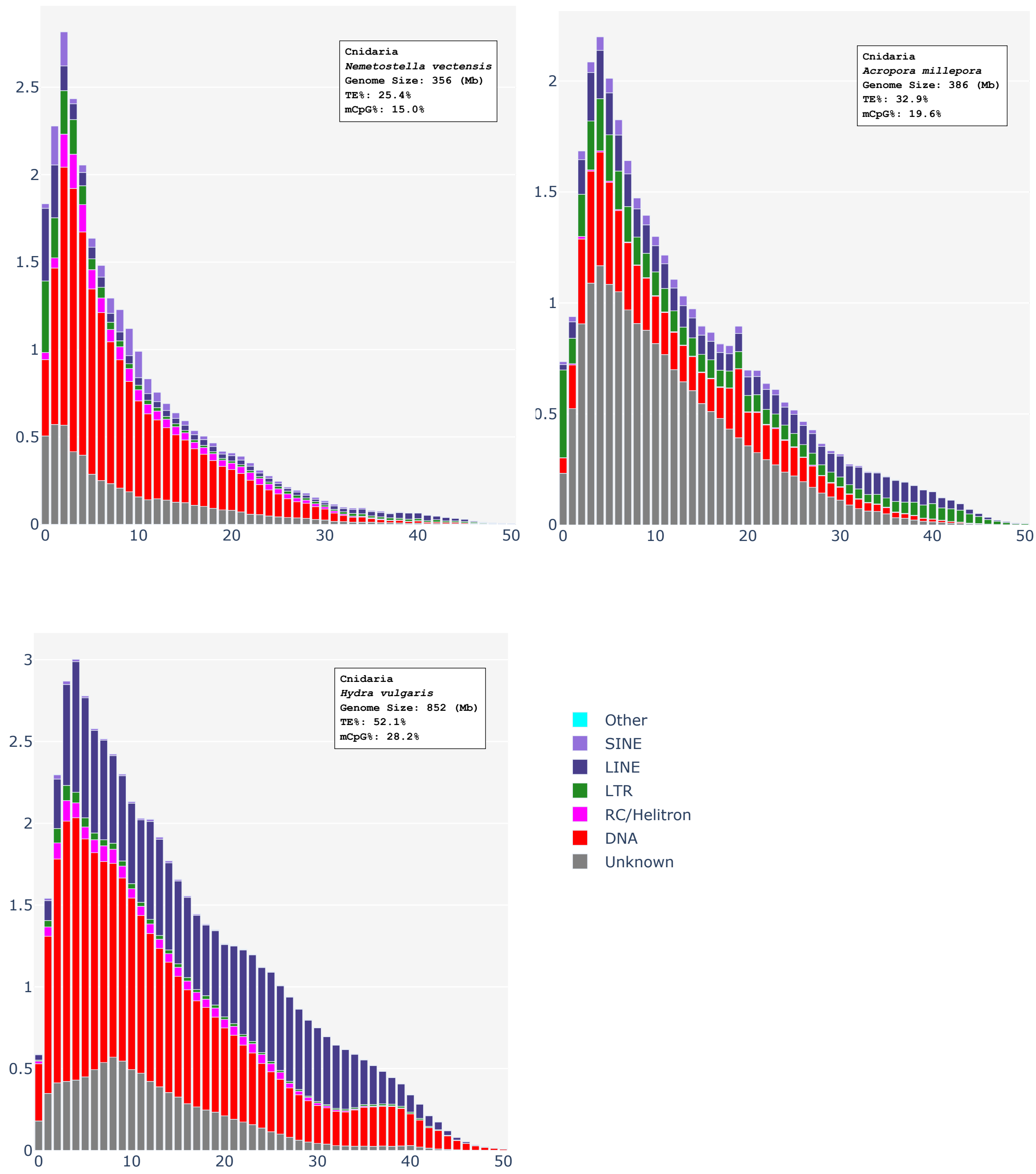

Supplementary Figure S1: Distribution of repeat classes and families at given divergence in the three cnidarian genomes. The x axis represents the extent of divergence (Kimura distance) of individual transposons from the corresponding consensus sequences generated by RepeatMasker. The y axis indicates the percentage of the genome accounted for.

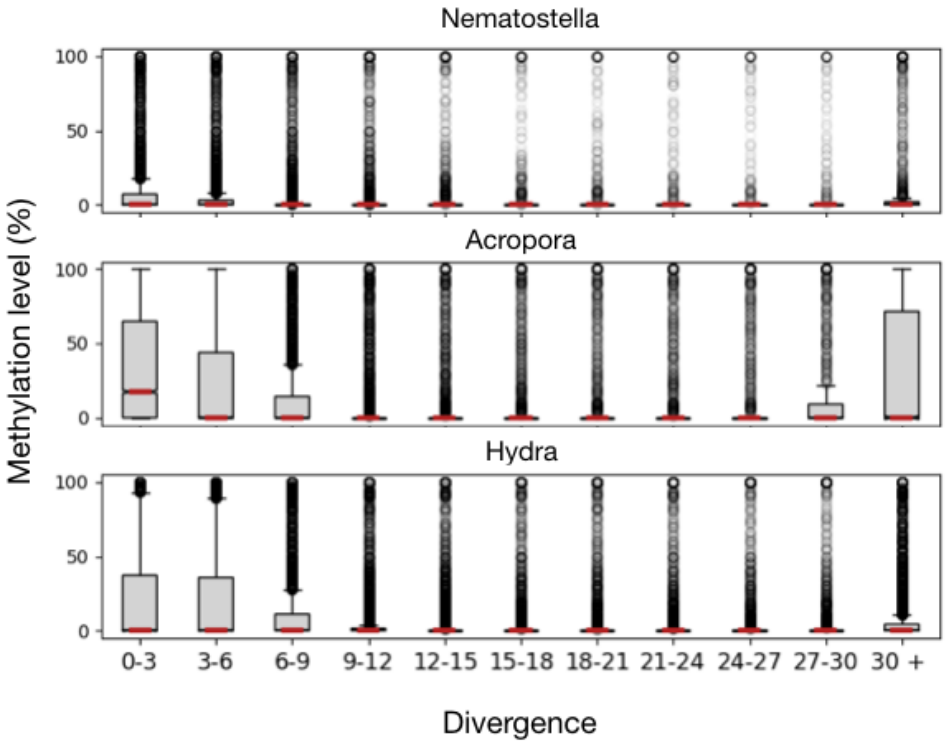

Supplementary Figure S2: DNA methylation level distribution of intergenic transposons across different divergence bins.

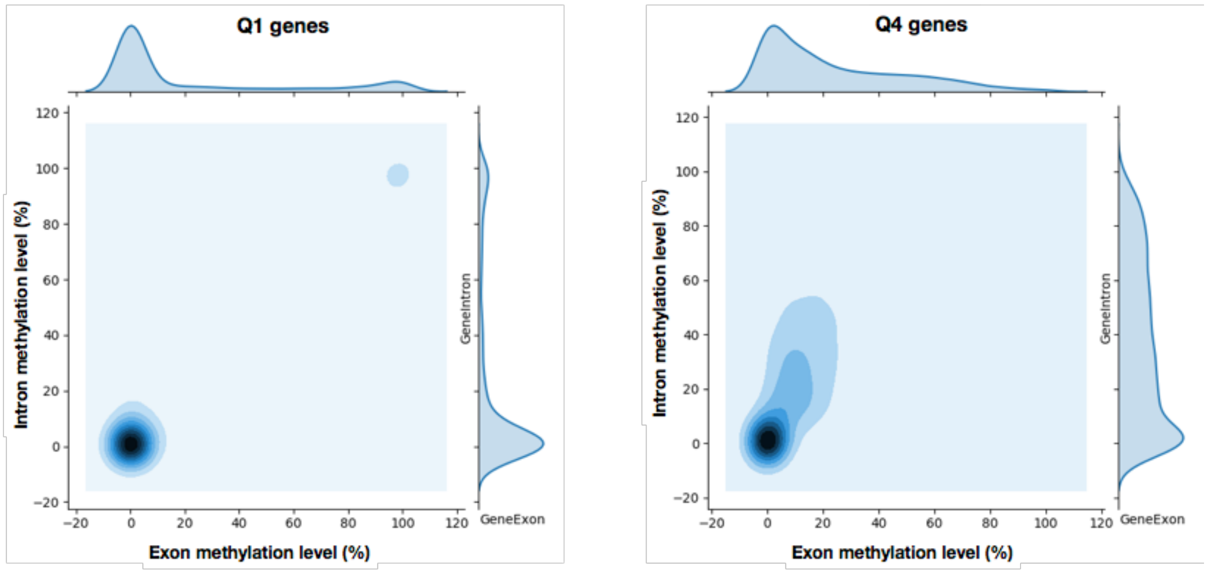

Supplementary Figure S3: Smooth kernel scatter plots showing that DNA methylation increases more in introns than in exons along with increases in gene expression levels. The x axis indicates exon ML and the y axis denotes the corresponding intron ML for each individual gene. For the lowest (Q1) expression quartile, both introns and exons tend to be either not methylated or almost fully methylated, and they agree with each other for a gene (along the diagonal). For the highest (Q4) expression quartile, the dark area is clearly shifted towards the upper part, which suggests that introns are more highly methylated than exons within the same gene.

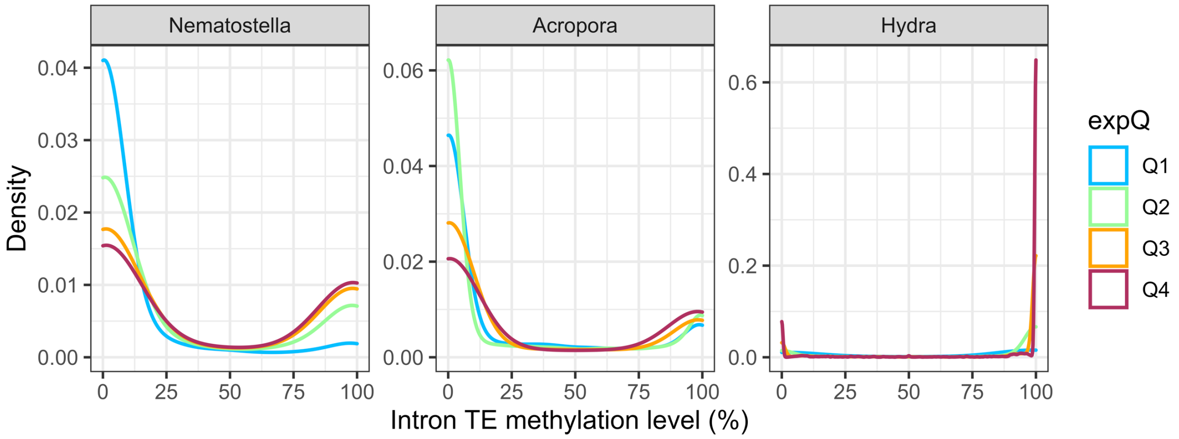

Supplementary Figure S4: Transposon methylation levels increase with gene expression level. The figure shows kernel density plots of transposon methylation level for transposons within gene bodies in genes across the lowest (Q1) to highest (Q4) expression quartiles across the three cnidarians.

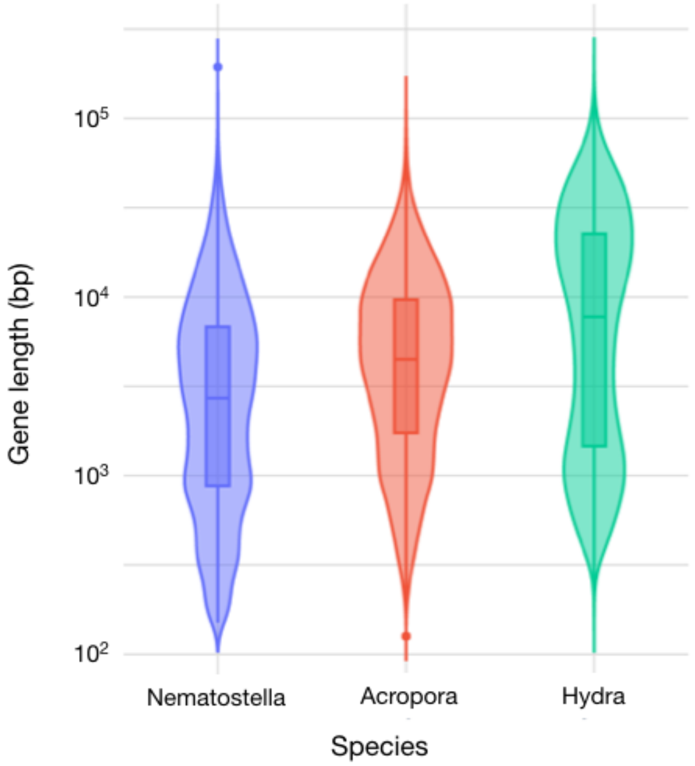

Supplementary Figure S5: Violin plots of gene lengths in the three cnidarian species. Although the sizes of the transcripts are similar across the three species (Supplementary table S8), *Hydra* genes are significantly longer; this size increase is a consequence of longer introns resulting from insertion of transposons.

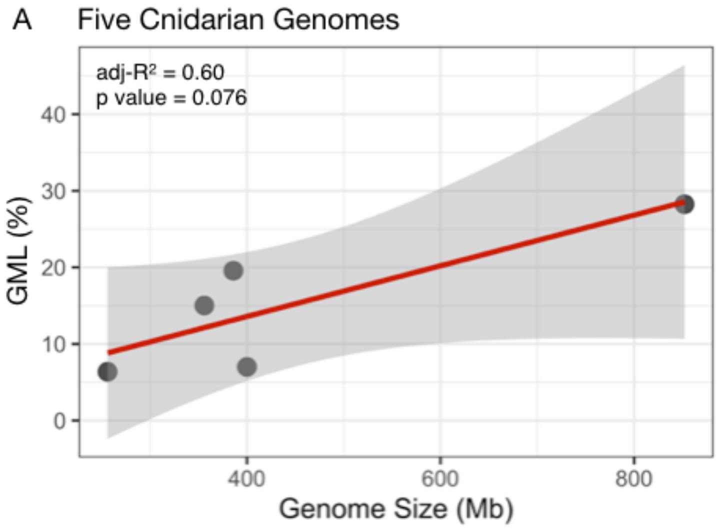

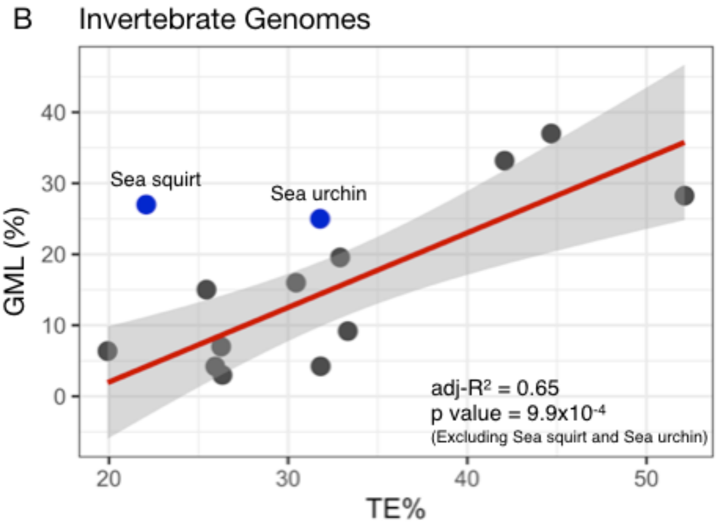

Supplementary Figure S6: (A) Relationship between genome size and transposon content in cnidarians. No correlation was observed ($p > 0.05$) across the same five cnidarian species as in Figure 6B. (B) Levels of genome wide methylation (y axis) and transposon content (x axis) for the same range of invertebrates as in Figure 6D, but also including the deuterostome invertebrates *Ciona* (a sea squirt) and *Strongylocentrotus* (a sea urchin). Note that the correlation shown was calculated after exclusion of the deuterostome data. Levels of methylation of the sea urchin and particularly the sea squirt genomes are somewhat higher than expected based on the transposon content, possibly as a consequence of secondary roles of methylation (in embryonic re-programming) in the deuterostome lineage (see text).
