# Supplementary material for: The Role of DNA Methylation in Genome Defense in Cnidaria and Other Invertebrates
Source: Mol Biol Evol. 2022 Jan 27;39(2):msac018. doi: 10.1093/molbev/msac018 (PMC8857917; doi:10.1093/molbev/msac018)
Supplement: msac018_Supplementary_Data [file msac018_supplementary_data.zip › MBE_Responses_20211222DM.docx]

**Reviewer 1:**

**Major comments:**

1. The authors could better highlight that they are considering single 'snapshots' of the methylation status of these animals, and whether or not they would expect this to affect their conclusions.

**Response:** We assume that the referee refers here to changes/differences in methylation levels occurring within a species during development, stress etc. These differences are much smaller than those between species (~ 10-fold differences) – within or very close to the estimated 95% confidence interval of mean GML for a given genome TE content in our analyses, and for this reason we are confident that our conclusions hold. To accommodate this point, the following text has been added to the Discussion: *While the DNA methylation levels presented are essentially snapshots of states that have been found to differ across a species range as well as between developmental stages and tissue types, these differences are small (usually 1-5%) compared to those between species (3-37% in this study). On this basis, such variations in GML within a species do not affect our conclusions.*

2. I would appreciate a short discussion on non-animal methylation and its role in transposon silencing e.g. in plants, fungi and what might be known about single celled holozoa to better establish that this is a plesiomorphic state.

**Response:** TE methylation is ubiquitous. Angiosperms show a similar pattern to what we report here, but employ different mechanisms. For example, MET1, a homolog of animal DNMT1, operates on CG methylation, while CHG methylation is maintained by the CMT3 gene. Niederhuth et al. 2016 (Figure 2A, <https://genomebiology.biomedcentral.com/articles/10.1186/s13059-016-1059-0>) observed stronger positive correlation between genome size and GML for CHG methylation than for CG methylation. Fungi don’t have gene body methylation, but do have TE methylation, as do some protists. To accommodate this point, the following text has been added to the Discussion: *Although mechanisms may differ, a positive correlation (between GML and genome size) is seen in flowering plants (Niederhuth et al. 2016); transposons are also targeted by methylation in fungi (Castanera et al. 2016) and some protists (Harony and Ankri 2008).*

**Minor comments:**

1. P.4 l.105 "hypermethylated" this is the first mention of hypermethylation, so the reader will not know what it is.

**Response:** At this point in the manuscript, we used the term “hypermethylated” to refer to significantly higher levels of methylation (on housekeeping genes relative to differentially expressed genes). A little later in the m/s, we define “hypermethylation” as ≥ 70% of CpGs being methylated. To avoid confusion, in the revised version of the m/s we replaced the first usage of “hypermethylated” with “highly methylated”.

2. P.5 l.145-6 - "including Exaiptasia pallida and Stylophora pistillata" needs references here as it is the first mention and otherwise location in results makes it appear as though done by authors.

**Response:** This was an oversight on our part, and has been modified as requested by the reviewer.

3. P.11 l.394 - "presumably leads to formation of localized heterochromatin-like structures" - it's not clear what the definition of "heterochromatin-like" is here, nor what the consequences are of presuming its formation. It seems to be an overreach into the molecular details, and I ask, does it matter?

**Response:** We used this wording because the documented phenomenon of a heterochromatin-like state imposed by methylation in plants essentially provides a precedent for our interpretation of the invertebrate data (i.e. “presumably leads to formation of localized heterochromatin-like structures, suggesting that localised methylation of transposons imposes a repressive state.”)

4. P.17 l.591 - legend talks about asterisks, but there are none in my copy of the figure.

**Response:** This was an error on our part (earlier versions of the figure were labelled with asterisks, but these were redundant) and has been corrected.

**Reviewer 2:**

**Comment:** To further support the claim that younger transposons are preferentially targeted by methylation, it maybe crucial to subdivide transposons according to their classes.

**Response:** We thank the referee for this suggestion, which is important because the three cnidarians do display different TE characteristics. After separating the TEs in each species into class I (retrotransposons) and class II (DNA transposons), younger transposons remained preferentially targeted by methylation in both introns and intergenic regions. This trend is also observed in the case of unclassified transposons. The figures below show the results for intron transposons. Although the overall trend is maintained, given the uncertainties arising from unclassified TEs (more detail is provided below), we felt that it would be unhelpful to include these plots in the manuscript.

In the revised version of the manuscript, the numbers of transposable elements in each divergence bin are listed in Supplementary table S7.

**Comment:** Kimura distances (from repeat consensus) can be misleading and they vary between different repeat classes. Similarly, the observation that there are many more younger repeats in Nematostella genome could be a result of assembly and/or repeat masking peculiarities which is worth noting. Since it is much likely to observe younger transposon insertions while older insertions are lost over time, it opens the question whether Nematostella is more capable of getting rid of ancient transposons than the other two cnidarians (thus being in a way faster evolving) or it just has less repeat activity in total.

**Response:** We wholeheartedly agree with the reviewer’s comments about the limitations that apply to the use of Kimura distances, although this is one of the most commonly used methods to infer transposon ages. A number of possible scenarios could account for loss (or apparent loss) of older insertions from the genome - for example genome reduction, which can ultimately eliminate old TEs completely. Old TEs may accumulate mutations to a point where they become inactive and undetectable, a high proportion of which likely lost DNA methylation over time. Unfortunately, as yet we are unable to address issues around transposon dynamics over evolutionary time and their variation across lineages.

**Comment:** In this respect, it maybe worth stating the absolute number of basepairs occupied by young vs old repeats in all three genomes. As a corroborative evidence for the young age of some repeats, authors may also include any of the available transcriptomic data to show that these elements are also transcriptionally active, which would not be expected to the same degree in the methylated ancient repeat group.

**Response:** Following this suggestion, in the revised version a new supplementary table (Table S7) has been added that provides the numbers of TE elements in each divergence bin and overall young versus old TE length for data used in Figure 3A and S2.

At this time, only limited data are available on transposon expression. In the case of Hydra, Chapman et al. 2010 mention that transcripts corresponding to CR1 non-LRT retrotransposons (which are the most abundant TE family and constitute 15% of the genome) and other (DNA and RNA) transposon families were detected in EST data. Clearly both retrotransposons and DNA transposons are active in Hydra, and we have detected transposon transcripts in Acropora data (e.g. Kortschak et al. 2003 Current Biology 13, 2190-2195) but to our knowledge no information available on levels of expression of transposons falling into different divergence classes.

While the issues raised by the referee are important and deserve further investigation, to comprehensively address these would require not only extensive additional analyses but also more data. This manuscript was based on a single RNA-seq library, but we consider that addressing (1) the relationship between transposon age (divergence) and transcriptional activity and (2) the impact of DNA methylation on this relationship would require BS-seq and RNA-seq data from multiple conditions with replicates – a substantial additional research project.

**Comment:** For correlative trends between the genome size/repeat content and the methylation levels it would be again good to have the graphs subdivided by repeat family or at least class, because we may expect different repeats showing distinct association with methylation in the three genomes.

**Response:** We appreciate the reviewer’s point – certainly, precedents imply that DNA transposons and retrotransposons may differ in terms of the host methylation response. However, unclassified transposons (unknown proportions of which belong to Class I and Class II) account for a major proportion of the total in all five cnidarians for which methylome data are available, and this compromises evaluation of the relationship between methylation level and transposon class. To address the referee’s comment, we first examined the relationship between TE classes and genome methylation level (GML) in the five cnidarian genomes, but found no significant correlation between any transposon class (retrotransposons, DNA transposon, or unclassified transposons) and GML:

We consider that the main cause of this result is – as mentioned above – the unclassified transposons issue. While transposons in the two model organisms (Nematostella and Hydra) are relatively well annotated, the proportion of unclassified transposons exceeds the combined proportion of retrotransposons and DNA transposons in the other three cnidarian genomes, particularly in the case of Exaiptasia. Consequently, we consider this result is unreliable.

Similarly, with the sole exception of Crassostrea, > 48% of total annotated transposons in the other invertebrates sampled in this study were unclassified. In summary, while such correlations are potentially both interesting and important to explore, the uncertainties arising as a consequence of large but variable proportions of unclassified transposons (which could belong to either class I or II) mean that these cannot be evaluated at present.

**Comment:** This trend may further be explored if the authors could also do similar correlations based on young (<10% divergence) and old (>30%) divergent repeat copies.

**Response:** Earlier (pre-submission) iterations of our manuscript explored this issue, but we decided not to include them because the sample size available to us for this study limited our ability to interpret the results explicitly.

We separated transposons into three age groups as Young (divergence < 10), Middle aged (10 ≤ divergence < 30) and Old (divergence ≥ 30). For the five cnidarian genomes, a positive trend was observed from all three TE age groups, but only young transposon content was significantly correlated with GML (p = 0.019).

Furthermore, it is intuitively preferable to apply a multiple regression model using the three age groups to better understand the relationship between GML and TE age. However, even for the 12 invertebrates, the sample size is still too small for three independent variables. While the overall trend is unchanged, the results defied simple interpretations – we don’t know whether this is due to model overfitting or something else, and feel that presenting these unclear results would add substantially to the length of the manuscript whilst contributing very little. Hopefully, in future this interesting question can better explored when more genome data are available.

**Comment:** This could be of interest to differentiate between the ancient cnidarian/metazoan traits and the derived characteristics of species-specific repeat expansion (e.g., in Hydra). Note that the 'ancient' >30% divergence level is estimated to span a relatively recent evolutionary time frame and would not capture the ancient cnidarian divergence. However, any old repeat families that show similar methylation regimes and that are shared between all three species could comprise an informative set for 'ancestral' characteristics worth of further investigation.

**Response:** The main conclusion of the paper is that DNA methylation plays a genome defense function. Because the species divergence times are so deep (for Acropora and Nematostella, which are the closest pair, > 400MY), any transposable elements that are shared between species are likely identifiable only because of exaptation. Once a transposon has accumulated certain numbers of mutations and become inactive, it should evolve in a neutral manner, which is the expected fate of the majority of transposons. Neutrally evolving DNA will lose sequence identity quite quickly, e.g. introns of rodents and primates do not show credible sequence homology over a much shorter evolutionary time-span. So, analyses of so-called shared "ancient" transposons will be strongly confounded by the influence of natural selection for the new function. To look for conserved transposons, the most informative approach may be to begin at a relatively shallow level – a number of closely related genomes could be mined to provide a set of TE sequences that can be aligned with confidence, before gradually moving to more deeply diverged species.
